# Phytopharmacology and Clinical Updates of *Berberis* Species Against Diabetes and Other Metabolic Diseases

**DOI:** 10.3389/fphar.2020.00041

**Published:** 2020-02-18

**Authors:** Tarun Belwal, Aarti Bisht, Hari Prasad Devkota, Hammad Ullah, Haroon Khan, Aseesh Pandey, Indra Dutt Bhatt, Javier Echeverría

**Affiliations:** ^1^Centre for Biodiversity Conservation and Management, G. B. Pant National Institute of Himalayan Environment and Sustainable Development (GBPNIHESD), Kosi-Katarmal, Almora, India; ^2^Department of Instrumental Analysis, Graduate School of Pharmaceutical Sciences, Kumamoto University, Kumamoto, Japan; ^3^Program for Leading Graduate Schools, Health Life Science: Interdisciplinary and Glocal Oriented (HIGO) Program, Kumamoto University, Kumamoto, Japan; ^4^Department of Pharmacy, Abdul Wali Khan University, Mardan, Pakistan; ^5^Centre for Biodiversity Conservation and Management, G.B. Pant National Institute of Himalayan Environment and Sustainable Development, Sikkim Regional Centre, Pangthang, Gangtok, India; ^6^Department of Environmental Sciences, Faculty of Chemistry and Biology, Universidad de Santiago de Chile, Santiago, Chile

**Keywords:** *Berberis*, berberine, diabetes, metabolic diseases, pharmacology, clinical studies

## Abstract

The incidences of diabetic mellitus and other metabolic diseases such as hypertension and hyperlipidemia are increasing worldwide; however, the current treatment is not able to control the rapidly increasing trend in diabetes mortality and morbidity. Studies related to the effectiveness of extracts and pure compounds obtained from plants have shown promising responses in preclinical and clinical studies related to these metabolic diseases. Plants belonging to the genus *Berberis* (Family: Berberidaceae) are widely distributed with nearly 550 species worldwide. Extracts and compounds obtained from *Berberis* species, especially Berberine alkaloid, showed effectiveness in the management of diabetes and other metabolic diseases. Various pharmacological experiments have been performed to evaluate the effects of *Berberis* extracts, berberine, and its natural and chemically synthesized derivatives against various cell and animal disease models with promising results. Various clinical trials conducted so far also showed preventive effects of *Berberis* extracts and berberine against metabolic diseases. The present review focuses on i) research updates on traditional uses, ii) phytopharmacology and clinical studies on *Berberis* species, and iii) active metabolites in the prevention and treatment of diabetes and other metabolic diseases with a detailed mechanism of action. Furthermore, the review critically analyzes current research gaps in the therapeutic use of *Berberis* species and berberine and provides future recommendations.

## Introduction

Diabetes mellitus (DM) is a metabolic disorder that is characterized by an abnormal long-term increase in plasma glucose levels. Diabetes is mainly classified into four types, i.e., type I diabetes (T1DM), type II diabetes (T2DM), gestational diabetes, and specific types of diabetes due to other causes (American Diabetes Association, 2019). Many factors, such as insulin deficiency or resistance as well as altered carbohydrate, protein, and fat metabolisms, are usually the reasons for high blood glucose levels leading to DM. Chronic hyperglycemia related to diabetes is often associated with many other complications, such as cardiovascular, dermatological, neurological, renal, retinal, and nerve diseases. Diabetes is one of the most common chronic disease, and it has shown an increasing rate of occurrence over the past decade (Bullard et al., 2018). According to the World Health Organization (WHO), the total number of people with diabetes worldwide substantially increased from 108 million in 1980 to 422 million in 2014 (World Health Organization, 2016). Along with diabetes, the incidence of other metabolic diseases, such as hyperlipidemia, is also increasing rapidly (Karr, 2017).

Metabolic syndrome (MS) is associated with a group of disease conditions that occur together, and it is composed of central adiposity, hyperglycemia, hypertriglyceridemia, low high-density lipoproteins (HDL)-cholesterol, and hypertension. This disease cluster of diabetes and cardiovascular diseases is also known as “The Deadly Quartet”, “Syndrome X”, and “The Insulin Resistance Syndrome” (Alberti, 2005). Various treatment options are available to mitigate MS, including the diabetic condition and related disorders (Deedwania and Volkova, 2005). As MS is manifested by the cluster of diseases, use of a single drug candidate might not be able to provide necessary therapeutic effects. Plant extracts and isolated compounds can be possible options as adjuvants in such cases. Traditionally, various medicinal plants and their products (extracts and isolated compounds) have been used in the treatment of diabetes and hypertension (Oyedemi et al., 2009; Tabassum and Ahmad, 2011; Rizvi and Mishra, 2013; Ezuruike and Prieto, 2014). Various research showed the protective/curative effect of plant extracts as a whole and/or an individual bioactive compound against diabetes and other metabolic diseases (Tabatabaei-Malazy et al., 2015; Waltenberger et al., 2016).

Plants belonging to the genus *Berberis* (Family: Berberidaceae) are widely distributed worldwide with nearly 550 species. A decoction prepared from the roots of *Berberis* plants is one of the common traditional recipes for the treatment of diabetes (Neag et al., 2018). Various studies have reported the traditional uses *Berberis* plants for the treatment of metabolic diseases (e.g., diabetes and hyperlipidemia) in many countries, including India, Pakistan, China, and Iran (Hamayun et al., 2006; Uniyal et al., 2006; Rahimi Madiseh et al., 2014; Rana et al., 2019). Various bioactive compounds, such as alkaloids, polyphenols, flavonoids, anthocyanins, etc., have been found in *Berberis* species along with various vitamins and mineral components (Andola et al., 2010; Srivastava et al., 2015; Belwal et al., 2016; Belwal et al., 2017). Berberine (BBR), a quaternary ammonium salt belonging to a group of benzylisoquinoline alkaloids, is the most active compound reported from *Berberis* species, and it is considered to be highly effective against diabetes and other metabolic diseases (Dong et al., 2012; Lan et al., 2015; Wang H. et al., 2018). BBR is also distributed in various plant species of other genera such as *Coptis*, *Hydrastis*, *Mahonia*, *Tinospora*, *Xanthorhiza*, and many others (Neag et al., 2018). In the genus *Berberis*, the distribution of BBR and other alkaloids is mostly in its root part, followed by the stem bark and the stem itself (Andola et al., 2010). In addition, its presence in trace amounts has been reported from leaves and berries. Various studies have been conducted to evaluate the effectiveness of *Berberis* extract or bioactive alkaloidal compounds against diabetes and other MS with promising results (Gulfraz et al., 2008; Meliani et al., 2011; Imenshahidi and Hosseinzadeh, 2016; Mirhadi et al., 2018). Moreover, various clinical trials were also conducted on testing their effectiveness against diabetes and other metabolic diseases and showed variable effects (Zhang et al., 2010; Pérez-Rubio et al., 2013).

Considering the *Berberis* species and their active alkaloidal components, the present review specifically focuses on their effectiveness against diabetes and other metabolic diseases. This review discusses various traditional uses of *Berberis* against metabolic diseases, along with its cell- and animal-model studies. The pharmacological effects of *Berberis* extracts and alkaloids against diabetes and other metabolic diseases are also discussed along with the molecular mechanism of action. Furthermore, based on the present studies of *Berberis* species against diabetes and metabolic diseases, research gaps were highlighted, and future recommendations were made.

## Methodology

The scattered scientific information on *Berberis* species and isolated compounds used to counteract metabolic diseases was collected and documented. The synonyms of the various species were crosschecked with the plant name database The Plant List (www.theplantlist.org, Retrieved on November 22, 2019). Afterwards, the available articles on respective species were retrieved using popular search engines and various databases, such as SciFinder, ScienceDirect, PubMed, Scopus, Mendeley, JOAP, Microsoft academic, and Google Scholar. The keywords used were *Berberis*, berberine, diabetes, metabolic diseases, metabolic syndrome, ethnopharmacology, ethnobotany, chemical constituents, alkaloids, *in vitro*, *in vivo*, clinical study, and clinical trials. The data were congregated through the Boolean information retrieval method by using a plant name along with an “AND” operator followed by diabetes and metabolic syndrome. No prerequisite limitations on publications, i.e., language, year, and publication type (original contribution, review article, or key editorial note), were taken into consideration.

## Taxonomy and Ecology of Genus *Berberis*

**Figure 1:**
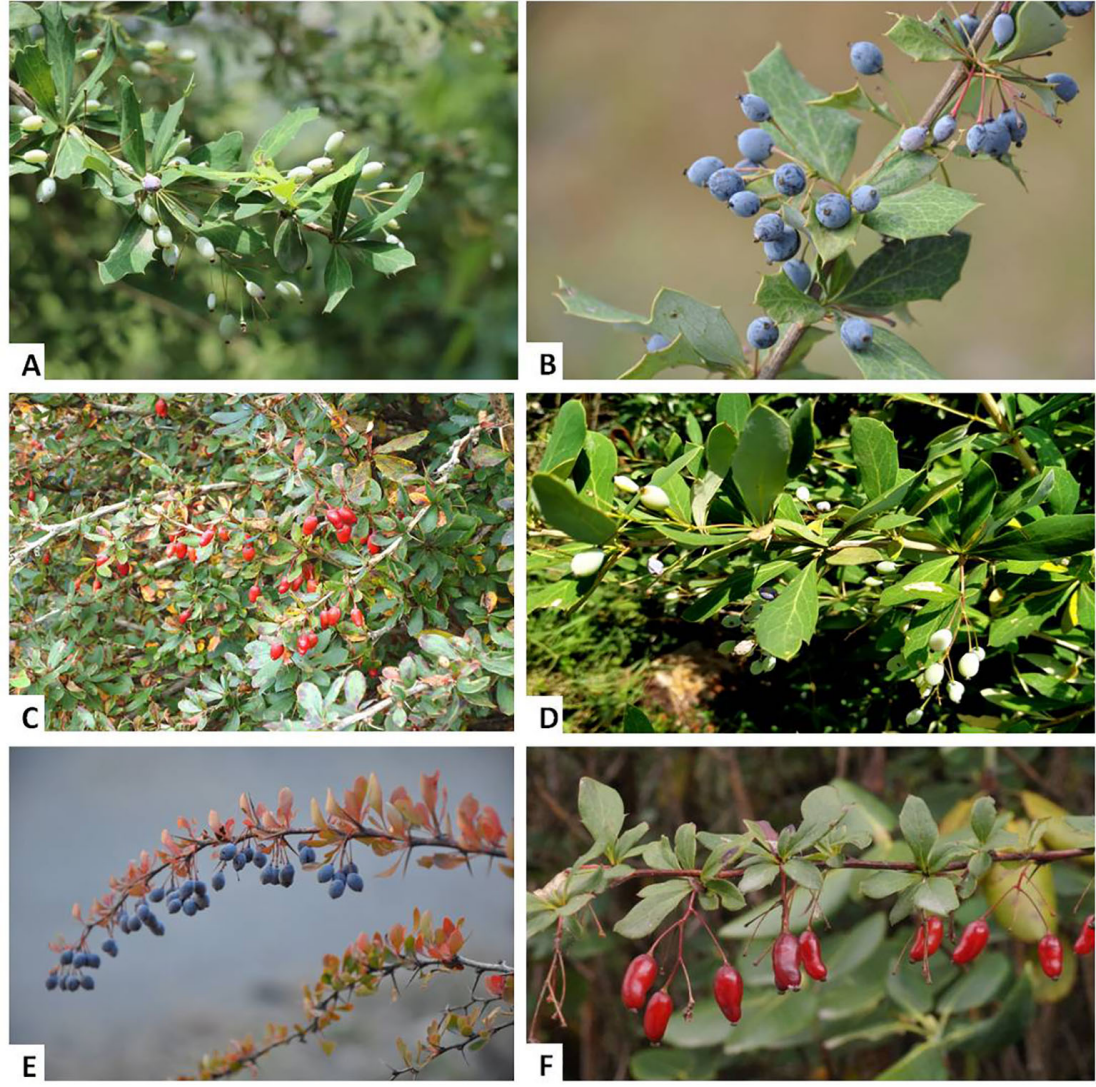
Some *Berberis* species of Indian Himalayan Region (IHR). **(A)** *B. aristata* DC., **(B)** *B. asiatica* Roxb. ex DC., **(C)** *B. jaeschkeana* C.K.Schneid., **(D)** *B. lycium* Royle, **(E)** *B. pseudumbellata* R. Parker, **(F)** *B. thomsoniana* Schneider.

**Figure 2:**
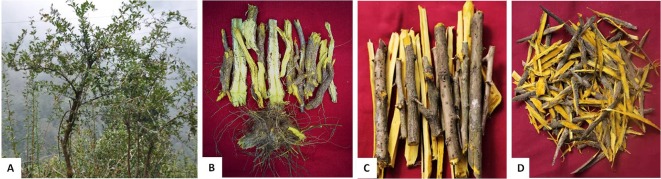
Various plant parts of **(A)** *Berberis asiatica* collected from Indian Himalayan Region (IHR), includes, **(B)** roots, **(C)** stems and **(D)** stem barks. These parts are the major sources to extract Berberine (yellow color) from *Berberis* species.

According to The Plant List database (www.theplantlist.org, retrieved on September 20, 2019), the family Berberidaceae consists of a total of 19 genera. The members of the genus *Berberis* are reported to be difficult to identify taxonomically due to their extreme morphological variation in relation to the environmental factors and natural hybridization (Ahrendt, 1961; Rao et al., 1998). Various overlapping morphological characters, such as flowers, leaves, stems, and berries—which also depend upon the season—and plant age also make it difficult to identify during field tasks (Rao and Hajra, 1993; Rao et al., 1998; Tiwari and Singh Adhikari, 2011). *Berberis* species are widely cultivated around the world due to their high medicinal and ornamental value. Most members of the genus *Berberis* are reported to be tolerant to shade, resistant to drought, and widely distributed in open and wooded habitats and wetlands. These plants are also studied as indicators of habitat degradation in the temperate region due traditionally to their thorny stem and unpalatable shoots (Champion and Seth, 1968). Representative photographs of some *Berberis* species from the Indian Himalayan Region (IHR) are shown in **Figure 1**, and their major plant parts used to extract berberine and other bioactive alkaloids are shown in **Figure 2**.

## Ethnopharmacology of *Berberis* spp. Against Diabetes and Other Metabolic Diseases

A literature review revealed that the ethnopharmacological uses of *Berberis* species have been documented from different parts of the world for the treatment of diabetes, hypertension, and obesity, and some of them also revealed the formulation methods. A majority of *Berberis* species were found to be used in the Himalayan region of India and Pakistan.

*B. lycium* Royle has been used traditionally for the treatment of diabetes mellitus and other diseases, particularly by the local inhabitants of the Himalayan region (Hamayun et al., 2006). Apart from diabetes, *B. lycium* is also used to treat bone fractures, diarrhoea, fever, intestinal colic, internal wounds, jaundice, menorrhagia, ophthalmic disorders, piles, rheumatism, sun blindness, and throat pain (Jabeen et al., 2015; Adhikari et al., 2019). Fruits and leaves of *B. lycium* are also reported to be used for the treatment of diabetes mellitus in south-west of Iran (Rahimi Madiseh et al., 2014) and Pakistan (Zain-Ul-Abidin et al., 2018). The water extract obtained by soaking the root bark in water is used for the treatment of diabetes (Ahmed et al., 2004). The whole plant is used to treat diabetes in Chamba district of Himachal Pradesh, West Himalaya, India (Rana et al., 2019). The Bhotiya tribal community of the Central Himalayan region of India used *B. lycium* roots with water for the treatment of diabetes (Phondani et al., 2010).

The stem of *B. aristata* DC. is widely used in Indian traditional medicine for the treatment of diabetes (Upwar et al., 2011), which is also reported in Ayurvedic Pharmacopoeia. The decoction (5–10 mL) of roots or stems of this species prepared with water was taken twice a day for 1–2 weeks to treat diabetes in Uttarakhand region (Kumar et al., 2019). It is also used by Uttarakhand people for the treatment of hypertension (Singh et al., 2019). The root, stem, and fruit also have been used to treat obesity (Chandrasekaran et al., 2018). *B. asiatica* is also used for the treatment of diabetes by the tribal communities of Chhota Bhangal, Western Himalaya, India. The decoction prepared from the roots is concentrated and dried in shade and then used with the sap of bitter guard for the treatment of diabetes (Uniyal et al., 2006).

In Iranian traditional medicine, *B. vulgaris* L. is extensively used to treat diabetes and hypertension (Rahimi-Madiseh et al., 2017). Local people use a decoction from the fruits and roots of *B. vulgaris* to treat hypertension (Baharvand-Ahmadi et al., 2016). The fruits are most frequently used in traditional and modern medicine (Rahimi Madiseh et al., 2014). Dried roots of *B. crateagina* DC. were recorded to be used as anti-diabetic agents locally in Turkey, and the decoction or infusion prepared from dried roots was taken orally one to two times a day for the treatment of diabetes (Durmuskahya and Öztürk, 2013). The anti-diabetic activity has also been reported for *B. brevissima* Jafri and *B. parkeriana* C.K.Schneid. (Alemardan et al., 2013). Bahmani et al. (2016) reported that the inhabitant of Urmia, Iran, use boiled and steamed *B. integerrima* Bunge extract for the treatment of diabetes.

## Alkaloids From *Berberis* Species: Potential Compounds Against Metabolic Diseases

**Figure 3:**
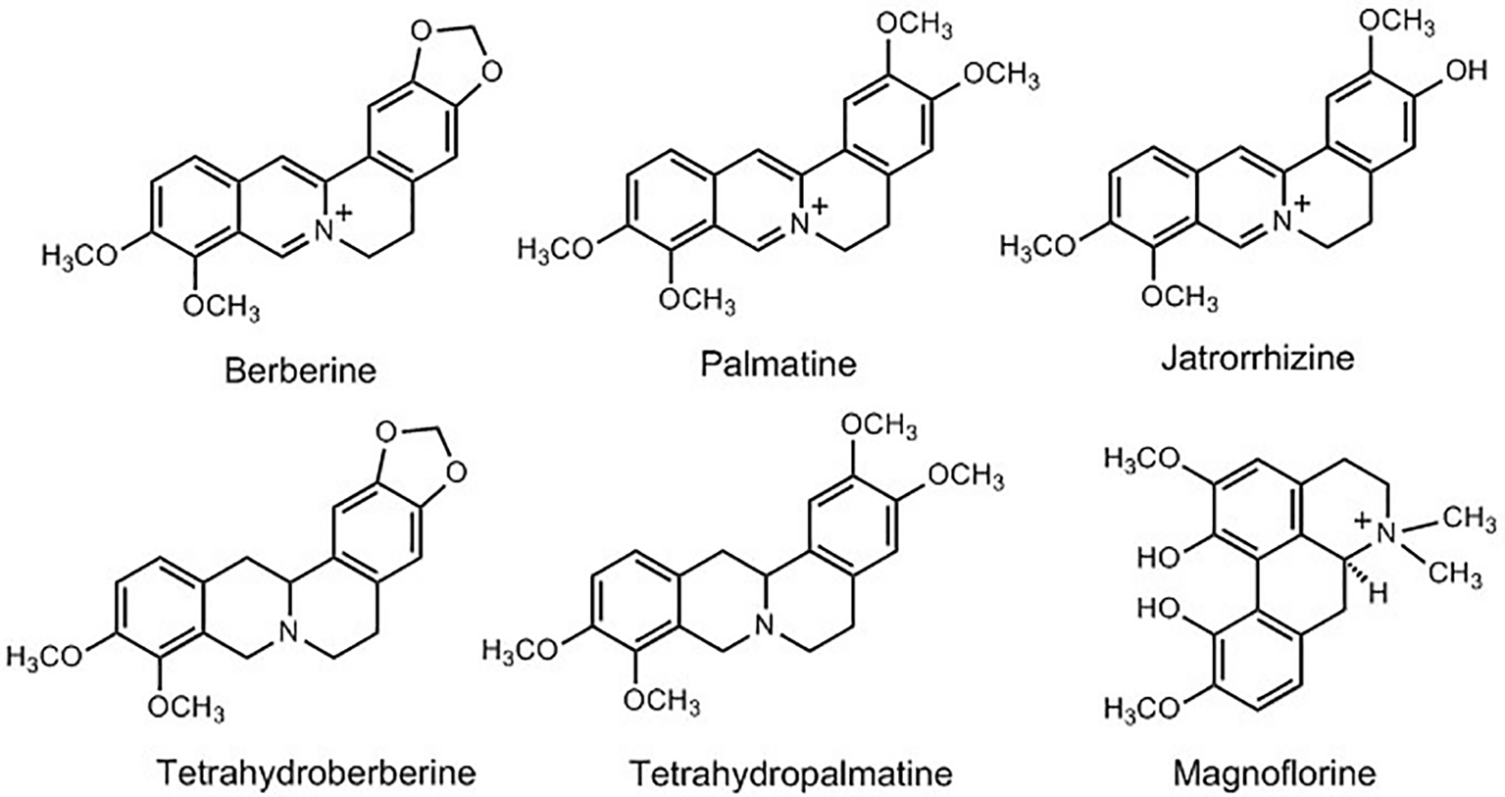
Structures of some of the main bioactive alkaloids from *Berberis* species.

A large number of studies have been conducted on the isolation and quantification of bioactive compounds from *Berberis* species. The phytochemical investigations of the genus *Berberis* have shown the presence of more than 105 compounds with varying structural confirmations. Most of the studies on *Berberis* species are focused on phytochemical screening; for the presence and estimation of different secondary metabolites, such as alkaloids, flavonoids, steroids, sugars, triterpenoids, tannins, and other preliminary assays such as total ash content, acid soluble ash content, and moisture content (Belwal et al., 2016; Belwal et al., 2017; Andola et al., 2018; Srivastava et al., 2006; Shahid et al., 2009). However, the isolation and characterization of alkaloids from genus *Berberis* is well documented. Alkaloids are one of the major bioactive chemical constituents of the *Berberis* species, and they are responsible for various pharmacological activities of either whole extract or isolated individual compounds. Berberine (BBR) is one of the most commonly reported alkaloids from various *Berberis* species along with palmatine, magnoflorine, and jatrorrhizine, etc. (**Figure 3**) (Bhardwaj and Kaushik, 2012; Feng et al., 2018). Simple isoquinolone alkaloids are mainly reported from these species; however, studies have also reported their dimmers or dimeric benzylisoquinoline alkaloids (Leet et al., 1983). The detailed list of different alkaloids isolated from various *Berberis* species are given in **Table 1**. Among other compounds, BBR and its various natural and synthetic derivatives have also been evaluated and found effective in prevention and treatment of MS (Pérez-Rubio et al., 2013; Li et al., 2015; Zhao et al., 2017).

**Table 1:** List of alkaloids isolated from various *Berberis* species.

Plant source	Plant parts	Alkaloids	References
*B. acanthifolium* Mart. ex Schult. & Schult.f.	Stem bark	Berberine, tetrahydropalmatine	(Tiwari and Masood, 1977)
*B. aetnensis* C. Presl.	Root	Berberine	(Alamzeb et al., 2015)
*B. amurensis* Rupr.	Stem	Berberine, palmatine, berberine	(Wu et al., 2015)
*B. amurensis* Rupr.	Young shoot	Berberubine, oxyacanthine, pseudopalmatine, amurenine	(Yusupov et al., 1993b)
*B. aristata* DC.	Stem bark	Berberine phenoxide, ketoberberine benzoate A, ketoberberine benzoate B	(Ahamad et al., 2014)
	Root and stem bark	Berberine, palmatine, berberrubine, jatrorrhizine, ketoberberine, dihydropalmatine, berbamine, pakistanamine	(Bajpai et al., 2015)
*B. asiatica* Roxb. ex DC.	Root	Berberine, oxyacanthine, berbamine, palmitine, jatrorrhizine, oxyberberine, tetrahydropalmatine, columbamine	(Bhakuni et al., 1968)
*B. baluchistanica* Ahrendt	Root	Pakistanine, pakistanamine, baluchistanamine, gandharamine	(Shamma et al., 1973; Shamma et al., 1974; Miana et al., 1979; Abu Zarga et al., 1982)
*B. buxifolia* Lam.	–	Chillanamine, (-)-osornine, (-)-curacutine, (-)-talcamine	(Leet et al., 1983)
*B. calliobotrys* Bien. ex Koehne	Root	Khyberine, pakistanamine, 1-O-methylpakistanine, pakistanine, chitraline, kalashine	(Fazal Hussain et al., 1980)
*B. chitria* Buch.-Ham. ex Lindl.	–	Berberine, palmatine, jatrorrhizine, oxyacanthine, *O*-methylcorydine-*N*-oxide	(Hussaini and Shoeb, 1985)
	Root bark	Palmatine,	(Choudhary et al., 2010)
*B. coletioides* Lechl.	-	Pronuciferine *N*-oxide, pronuciferine	(Fajardo et al., 2009)
*B. concinna* Hook.f.	Stem bark	Berberine, tetrahydropalmatine	(Tiwari and Masood, 1977)
*B. crataegina* DC.	Stem and root	Berberine, palmitine	(Petcu, 1968)
	Seed	Berbaine, oxyacanthine	
*B. darwinii* Hook.	–	magallanesine	(Valencia et al., 1985)
*B. densiflora* Boiss. & Buhse	Leaf	Berberine, β-allocryptopine, densinine, densiberine, glaucine, oxyacanthine, thalicmidine, isocorydine, *O*-methylcorypalline	(Khamidov et al., 1997c)
*B. diaphana* Maxim.	Bark	Berberine, palmatine, magnoflorine, jatrorrhizine	(Feng et al., 2018)
*B. dictyophylla* Franch.	Bark	Berberine, palmatine, magnoflorine, jatrorrhizine	(Feng et al., 2018)
*B. glaucocarpa* Stapf	Root	Oxyacanthine, tetrandrine	(Alamzeb et al., 2018)
*B. heterobotrys* E.L.Wolf	–	Berberine, palmatine, yatrorizine, oxyacanthine, berbamine, reticuline, obaberine, isocorydine, talikmidine, berberal.	(Karimov et al., 1993b)
*B. heteropoda* Schrenk	Young shoot and leaf	*N*-Methyldihydroberberine, 8-oxoberberrubine, berbamunine, aromoline, glaucine, talikmidine, isocorydine, reticuline, Pseudopalmatine, laudanosine, berpodine, isotetrandrine	(Karimov et al., 1992; Karimov et al., 1993a; Yusupov et al., 1993a)
*B. hispanica* Boiss. & Reut.	Root bark	Berberine tannate	(Aribi et al., 2017)
*B. ilicifolia* L.f.	–	Ilicifoline	(Fajardo et al., 1996)
*B. iliensis* Popov	Young shoot	(+)-β*-N*-Methylcorypalmine, berberrubine, berberine, magnoflorine	(Karimov and Shakirov, 1993)
*B. integerrima* Bunge	–	Berberine, berbamunine, oxyacanthine, magnoflorin, intebrine, intebrinine, intebrimine	(Karimov et al., 1977; Karimov et al., 1993g; Karimov et al., 1993h)
*B. jaeschkeana* C.K. Schneid	Root and bark	Berberine	(Andola et al., 2018)
*B. jaeschkeana* Schneid var. *jaeschkeana*	–	Berberine, palmatine, jatrorrhizine, chondrofoline, berberidione	(Alamzeb et al., 2015)
*B. julianae* C.K. Schneid.	Aerial part	Berberine, magnoflorine, glaucine, tetrahydrojatrorrhizine	(Brazdovicova et al., 1975)
*B. kansuensis* Schneid.	Bark	Berberine, palmatine, magnoflorine, jatrorrhizine	(Feng et al., 2018)
*B. laurina* Thunb.	Leaf	Berberine, (-)-tetrahydropalmatine, protopine	(Falco et al., 1968)
	Trunk bark and root	Berberine, obaberine (*O*-methyloxyacanthine), *O*-methylisothalicaberine, lauberine	(Falco et al., 1968)
*B. libanotica* Ehrenb.	Root, fruit	Oxycanthine, berbamine, jatrorrhizine, palmatine, berberine	(Alamzeb et al., 2015; Hosry et al., 2016)
*B. lycium* Royle	Fruit	Berberine, magnoflorine	(Sharma et al., 2018)
	–	Berberine, berbericine	(Sehdev et al., 1971)
*B. nummularia* Bunge	Leaf	Bernumine bernumidine and bernumicine, nummularine	(Karimov et al., 1993d; Faskhutdinov et al., 1997)
*B. oblonga* Scheid	Leaf	Glaucine, hydroxyacanthin, berbamine, berberin, isocoridin	(Khamidov et al., 2003)
	–	Berberine, berbamunine, oxyacanthine, magnoflorine, palmitine, oblongamine	(Karimov et al., 1977)
	Root	Berberine iodide, magnoflorine iodide, columbamine iodide, oxyacanthine, berbamine, 2'-*N*-methylisotetrandrine iodide	(Karimov and Lutfullin, 1986)
	Leaves and shoots	Thalicmidine and in the shoots, berberin. Other alkaloids isolated included glaucine, hydroxyacanthine, berbamine, isocoridine	(Khamidov et al., 2003)
*B. pachycantha* Koehne	Whole plant	Pachycanthine	(Ahmed et al., 2008)
*B. petiolaris* Wall. ex G. Don	Fruits, leaf, root and stem	Berberine, palmatine, magnoflorine, jatrorrhizine, tetrahydropalmatine, tetrahydroberberine, thalifendine/berberrubine, demethyleneberberine, reticuline, 8-oxoberberine, *N*-methyltetrahydroberberine,	(Singh et al., 2015)
	Root	Berbamine, berberine chloride, palimitine	(Miana and Ikram, 1970)
*B. sibirica* Pall.	Aerial part	(-)-Tetrahydropseudocoptisine, pseudoprotopine, (+)-chelidonine, (+)-glaziovine, berberine, palmatine, columbamine, berberubine, oxyacanthine, berbamine, 8-oxoberberine, 8-oxoberberubine, pakistanine, pronuciferine, *N*-acetylhomoveratrylamine	(Karimov et al., 1993e; Istatkova et al., 2007)
*B. tabiensis* L.A. Camargo	Stem	Tabienine	(Quevedo et al., 2008)
*B. thunbergii* DC	Stem	Berberine, berbamine, glaucine, isocorydine, oxycanthine, palmatine, thalicmidine	(Khamidov et al., 1997a)
	Leaf	Thalicmidine, oxycanthine, isocorydine, heliamine, berberine	(Khamidov et al., 1997a)
	Fruit	Oxyxanthine, isotetrandrine, thalicmidine	(Khamidov et al., 1997a)
	–	Berberine, columbamine	(Och et al., 2017)
	–	Oxyacanthine, palmatine, thalicmidine, isotetrandrine, berberine, berbamine, glaucine, isocorydine,heliamine	(Khamidov et al., 1997b)
*B. turcomanica* Kar. ex Ledeb.	Young shoot	Turconidine	(Karimov et al., 1993f)
	–	Turcberine	(Karimov et al., 1993c)
	Young shoot	Berberine, isocorydine, glaucine, thalicmidine, aromoline, oxyacanthine, turcomanine, berberine, papaverine, cyclotriveratrilene	(Khamidov et al., 1996d; Khamidov et al., 1996a)
	Leaf	Turcomanidine, Turcamine,	(Khamidov et al., 1996b; Khamidov et al., 1996c)
*B. vernae* Schneid.	Bark	Berberine, palmatine, magnoflorine, jatrorrhizine	(Feng et al., 2018)
*B. virgetorum* C.K. Schneid.	Whole plant	(-)-Berbervirine, berberine, jatrorrhizine, noroxyhydrastinine	(Liu et al., 1995)
*B. vulgaris* L.	Root bark	Berberine, palmatibne, bersavine, muraricine, berbostrejdine, berbamine, aromoline, obamegine, 8-oxoberberine, berbidine, bargustanine, Berberine, oxyacanthine, talikmidine, yatrorizine, berbamine, berbamunine, isocorydine	(Karimov et al., 1993i; Khamidov et al., 1995; Hošt'álková et al., 2013; Hostalkova et al., 2019)
*B. vulgaris* subsp. *australis* (Boiss.)	Root bark	Berbamine, sotetrandrine, oxyacanthine, obaberine, aromoline, obamegine, thaligrisine, thalifoline, 8-oxyberberine, chilenine, (-)-tejedine	(Suau et al., 1998)

The effect of different habitat conditions (altitudinal variations and edaphic factors) of *Berberis* species has been investigated. Chandra and Purohit (1980) investigated eight *Berberis* species from different altitudinal range for determining the BBR concentration in different parts. Among these, *B. asiatica* was found to contain higher content of BBR than other species. Lower altitudinal range was found to contain higher BBR content within a species as compared to high altitude habitat. Among plant parts, roots contained a higher concentration of BBR (Chandra and Purohit, 1980). Similarly, variations in the BBR content of five *Berberis* species (i.e., *B. aristata, B. asiatica, B. jaeschkeana, B. lycium*, and *B. pseudumbellata*) depending upon the habitat have also studied. The presence of higher BBR content was recorded from rocky habitats in *B. jaeschkeana* (Andola et al., 2018). Both altitude and edaphic conditions were found to be responsible for the variation in BBR content in root and stem bark. Lower altitude populations showed significantly higher BBR content and positively correlated with moisture and potassium availability in soil species. Among these, *B. asiatica* contain significantly higher BBR content as compared to other species (Andola et al., 2010) Seasonal variations in the BBR content revealed higher percentage in summer and lower in rainy season (Andola et al., 2018). Low moisture and high soil potassium level is reported to be well correlated with high BBR content (Andola et al., 2011).

## *In Vitro* Activities Against Diabetes and Other Metabolic Diseases

It has been suggested that physical exercise and a proper diet can act as controllers of the cause of T2DM and metabolic diseases. Currently available pharmacological interventions can control many aspects of diabetes and metabolic diseases, like microvascular and macrovascular complications, hypertension, dyslipidemia, and obesity. However, there is also a need for novel therapeutic agents that work alone or in combination with currently available drugs. Within the pharmacological options, phytochemicals have a great potential to act against T2DM, MS, and associated complications (Davì et al., 2010). Extracts of *Berberis* species and their components, especially alkaloids, have been documented for their potential activity against T2DM and MS in various *in vitro* studies (**Table 2**) (Potdar et al., 2012)

**Table 2:** *In vitro* activity of extracts and/or isolated compounds from *Berberis* species against diabetes and metabolic diseases.

Extracts from *Berberis* spp./isolated compounds	Model	Outcomes	**References**
Berberine			
Berberine (BBR)	Mouse 3T3-L1 cells	Downregulated transcription factors (CCAAT/enhancer binding protein β, CCAAT/enhancer binding protein α) and PPARγ, suppress PPARs, A-FABP and FASN and inhibit 3T3-L1 fibroblast differentiation to adipocytes	(Kishimoto et al., 2015)
Berberine (BBR)	Mitochondria isolated from the liver of high-fat-fed rats	↓capacity to accumulate calcium and OXPHOS capacity (MMP, oxygen consumption, and cellular ATP levels). ↑ mitochondrial SirT3 activity, normalizing mitochondrial function, and preventing a state of energetic deficit caused by impaired OXPHOS	(Teodoro et al., 2013)
Berberine (BBR)	C2C12 cell line	Reverted mitochondrial dysfunction induced by HFD and hyperglycemia in skeletal muscle, in part due to an ↑ in mitochondrial biogenesis. The prevention of mitochondrial dysfunction, ↑ in mitochondrial biogenesis, and BBR-induced AMPK activation, are blocked in cells in which SIRT1 has been knocked down.	(Gomes et al., 2012)
Berberine (BBR)	Cultured human liver and L6 rat skeletal muscle cells	↑ InsR mRNA and ↑ protein expression in dose- and time-dependent results. InsR expression in the L6 rat skeletal muscle cells. BBR-enhanced InsR expression improved cellular glucose consumption only in the presence of insulin. Silencing InsR gene with small interfering RNA or blocking the pi3k ↓ this effect. BBR-induced InsR gene expression through a PKC-dependent activation of its promoter. Inhibition of PKC abolished BBR-caused InsR promoter activation and InsR mRNA transcription.	(Kong et al., 2009)
Berberine (BBR)	3T3-L1 preadipocytes	Inhibitor of PPARγ and α	(Huang et al., 2006)
Berberine (BBR)	Human platelet	Inhibited platelet aggregation, superoxide production *via* modulating AR, NOX, and glutathione reductase activities in HG	(Paul et al., 2019)
Berberine (BBR)	Primary hepatocytes	Promotion of glucose uptake and prevention of gluconeogenesis by inhibition of SIRT3, and by regulation of mitochondria-related pathways.	(Zhang et al., 2018)
Berberine (BBR)	HepG2 and mouse primary hepatocytes	Prolonged activation of AMPK BBR-induced ↑CD36 expression in hepatocytes, evoking in FA uptake *via* processes associated to hepatocellular lipid accumulation and fatty liver.	(Choi et al., 2017)
Berberine (BBR)	H9c2 cardiomyocytes	Attenuation of palmitate-induced reduction in glucose uptake and consumption by ↓cellular DAG levels and accumulation of TAG.	(Chang et al., 2016)
Berberine (BBR)	Rat MCs	Inhibition of mesangial cell proliferation and hypertrophy by modulating cell cycle progress. Suppression of high glucose-induced TGF-β1 and FN expression through blocking NF-κB/AP-1 pathways.	(Lan et al., 2014)
Berberine (BBR)	human hepatoma cells	Upregulated LDLR expression independent of sterol regulatory element-binding proteins, but dependent on ERK activation. Also ↑ LDLR expression through a post-transcriptional mechanism that stabilizes the mRNA.	(Kong et al., 2004)
Berberine (BBR)	Omental adipose tissue biopsies	Inhibition of human preadipocyte differentiation and leptin and adiponectin secretion accompanied by downregulation of PPARγ2, C/EBPα, adiponectin, and leptin mRNA expression	(Yang et al., 2012)
Berberine (BBR)	3T3-L1 adipocytes, L6 myotubes, and L6 cells	↑AMPK in 3T3-L1 adipocytes and L6 myotubes, ↑GLUT4 translocation in L6 cells in a pi3k -independent manner, and ↓ lipid accumulation in 3T3-L1 adipocytes	(Lee et al., 2006)
Berberine (BBR)	CEM, HCT-116, HepG2.2.15, SW1990, HT1080 and 293T cell lines	↑gene expression of the insulin receptor	(Zhang et al., 2010)
Berberine (BBR)	L929 cells	Activation of GLUT 1 transporter	(Cok et al., 2011)
Berberine (BBR	3T3-L1 and L6 cells	Inhibition of PTP1B, and ↑IR and ↑IRS1 phosphorylation	(Chen et al., 2010)
Berberine (BBR)	3T3-L1 cells	↓TG accumulation by ↑pIRS1-PI3KpAkt, ↑GLUT4 translocation and ↑insulin tropic action by pCREB-pIRS2-pAkt	(Ko et al., 2005)
Berberine (BBR)	L6 cells	↑AMPK and ↑p38 MAPK phosphorylation	(Cheng et al., 2006)
Berberine (BBR)	3T3-L1 cells	Regulation of PPARs and positive transcription elongation of factor b expression	(Zhou and Zhou, 2010)
Berberine (BBR)	HepG2 and C2C12 cells	↑glucose metabolism by glycolysis stimulation and mitochondrial respiratory chain inhibition	(Xu et al., 2014)
Berberine (BBR)	HL-7702, normal human liver cell lines	LDLR up-regulation by AMPK-dependent Raf-1 activation	(Li et al., 2014)
**Combination of berberine and/or derivatives**			
Berberine (BBR) and dihydroberberine	L6 and LKB1−/− cells	AMPK activation, by complex I inhibition of the mitochondrial transport chain	(Turner et al., 2008)
9-*O*-lipophilic group substituted) berberine (9-*O*-BBR)	HepG2 cells	↑ hypoglycemic activity	(Zhang et al., 2016)
13-Methylberberine (13-Me-BBR)	Mouse 3T3-L1 cells	Downregulated the expression of adipocyte differentiation transcription factors (PPARγ and C/EBPα). ↓PPARγ, ↓C/EBPα, and ↓SREBP-1 protein levels. Effect require AMPK signaling pathway	(Chow et al., 2016)
Berberine (BBR) and metformin	HepG2 hepatocytes and C2C12 myotubes	Promotion of glucose metabolism *via* stimulation of glycolysis, not be related to AMPK activity.	(Xiao et al., 2018)
BBR derivatives: thalifendine	Human HepG2 liver cells	↑LDLR or InsR protein expression.	(Wang et al., 2009)
BBR amide derivatives	HL-7702 cells	↑ glucose-lowering efficacies	(Ren et al., 2017)
Mannose modified berberine (m-BBR)	HepG2 cells	↑ antidiabetic activity	(Han et al., 2019)
Pseudoberberine (pBBR)	HepG2 cells	AMPK activation and LDR up-regulation.	(Wang et al., 2012)
Palmatine	Differentiated myocytes, L6 cells	anti-diabetic activity may be mediated through insulin dependent pathway by the activation of IRTK and PI3K	(Sangeetha et al., 2013)
***Berberis* extracts**			
*B aristata* bark methanolic extract	Dipeptidyl peptidase IV	Inhibition of dipeptidyl peptidase IV activity	(Chakrabarti et al., 2011)
*B. mycrophylla* roots ethanolic extract	non-resistant and insulin-resistant HepG2 cells	hypoglycemic effects and ↑ glucose uptake by activating AMPK protein.	(Furrianca et al., 2017)
*B. vulgaris* roots (ethanolic extract) and berberine (BBR)	α-Glucosidase	↑ α-glucosidase activity, extract > BBR	(Abd El-Wahab et al., 2013)
*B. vulgaris* roots (methanolic extract)	α-Amylase	↑ α-amylase activity	(Boudjelthia et al., 2017)
Jinqi Jiangtang tablet (berberine-contain)	α-Glucosidase, lipase and aldose	↑α-glucosidase, ↑lipase, and ↑aldose reductase activities,	(Chang et al., 2015)

The ↑ and ↓ signs shows significant increase and significant decrease of evaluated factors during mentioned studies.

Studies in mouse 3T3-L1 cells suggested that BBR has an pivotal role in regulating adipose tissues (Kishimoto et al., 2015). Experiments in mitochondria isolated from the liver of high-fat-fed rats have shown that BBR exhibited protective effects against MS that was associated with the increased mitochondrial sirtuin-3 (SIRT3) activity, normalizing mitochondrial function, and preventing a state of impaired oxidative phosphorylation (OXPHOS) that caused energetic deficit (Teodoro et al., 2013). In the same way, the preventive effects of BBR on diet-induced insulin resistance (InsR) was suggested to be linked to sirtuin-1 (SIRT1) and mitochondrial biogenesis (Gomes et al., 2012). It has been suggested that BBR is a unique natural medicine against insulin resistance in T2DM and MS (Kong et al., 2009). Different investigations have concluded that BBR as a new hypolipidemic drug works by a different mechanism of action to that of statin drugs (Kong et al., 2004). BBR works on multiple molecular targets as an inhibitor of peroxisome proliferator-activated receptor (PPAR) γ and α and is a potential weight reducing, hypolipidemic, and hypoglycemic agent (Huang et al., 2006). Prolonged activation of AMP-activated protein kinase (AMPK) by BBR improved CD36 expression in hepatocytes and was evoked in fatty acid uptake *via* processes associated with hepatocellular lipid accumulation (Choi et al., 2017). Also, BBR improved insulin sensitivity (InsS) by inhibiting fat storage and adjusting the adipokine profile in human preadipocytes (Yang et al., 2012). The hypoglycemic effects of BBR have also been attributed to its acute activation of the transport activity of glucose transporter 1 (GLUT1) (Cok et al., 2011).

Numerous studies of BBR in *in vitro* models have shed light on its positive effect on T2DM. BBR promoted glucose uptake and inhibited gluconeogenesis by inhibiting SIRT3, and regulating the mitochondria-related pathways (Zhang et al., 2018). BBR treatment attenuated a palmitate-induced reduction in glucose uptake and consumption through a reduction in cellular diacylglycerol (DAG) levels and the accumulation of triacylglycerol (TAG) in H9c2 cells (Chang et al., 2016). In addition, BBR displayed beneficial effects in the treatment of diabetes and obesity *via* stimulation of AMPK activity (Lee et al., 2006). The mechanisms of action of BBR in treatment of T2DM are suggested to be different than that of metformin and rosiglitazone (Zhang et al., 2010). BBR, as an insulin signal activator, had shown insulin-mimicry effects through the inhibition of protein tyrosine phosphatase 1B (PTP1B) activity on both adipocytes and myocytes (Chen et al., 2010) and acted as an effective insulin sensitizing and insulinotropic agent (Ko et al., 2005). Moreover, BBR and metformin promoted glucose metabolism by stimulating glycolysis through the inhibition of mitochondrial respiratory chain complex I and independent of AMPK activation (Xu et al., 2014). Besides, BBR circumvented the insulin signaling pathways and stimulated the glucose uptake through the AMP-AMPK-p38 MAPK pathway (Cheng et al., 2006). BBR modulated metabolism-related PPARs expression and differentiation-related positive transcription elongation factor b (P-TEFb) expression in adipocytes, which are associated with its hypoglycemic and hypolipidemic effects (Zhou and Zhou, 2010). In addition, BBR upregulated LDL receptor expression through Ras-independent (but AMPK-dependent) Raf-1 activation in liver cells (Li et al., 2014). BBR and metformin induced glycolysis and glucose consumption but are not related to the AMPK status (Xiao et al., 2018).

Different natural and synthetic derivatives of berberine are also evaluated for their *in vitro* activities. A BBR derivative, thalifendine, showed upregulatory activities for both LDLR and InsR, proving to be a potential treatment of both hyperlipidemia and hyperglycemia (Wang et al., 2009). Similarly, BBR amide derivatives improved the glucose-lowering effects (Ren et al., 2017). Mannose-modified BBR derivative exhibited high anti-diabetic activity at both high and low drug concentrations (Han et al., 2019). Palmatine showed anti-diabetic activity mediated through an insulin-dependent pathway by the activation of IRTK and PI3K (Sangeetha et al., 2013). Pseudoberberine (pBBR) has exhibited a potential effect on AMPK activation and LDLR upregulation as compared with BBR (Wang et al., 2012).

In the same way, the effects of extracts of species of the genus *Berberis* have been studied in several *in vitro* models and found effective. For instance, *B. mycrophylla* root extracts showed hypoglycemic effects and stimulated glucose uptake in HepG2 cells with and without resistance by activating AMPK protein (Furrianca et al., 2017). *B. aristata* bark methanolic extracts also inhibited the dipeptidyl peptidase–IV (DPP-IV) enzyme activity (Chakrabarti et al., 2011). *B. vulgaris* roots (ethanolic extract) and BBR showed α-glucosidase inhibition, where the inhibition caused by the extract was found to be higher than that of the BBR alone (Abd El-Wahab et al., 2013), and the extract also showed α-amylase inhibition activity (Boudjelthia et al., 2017).

**Figure 4:**
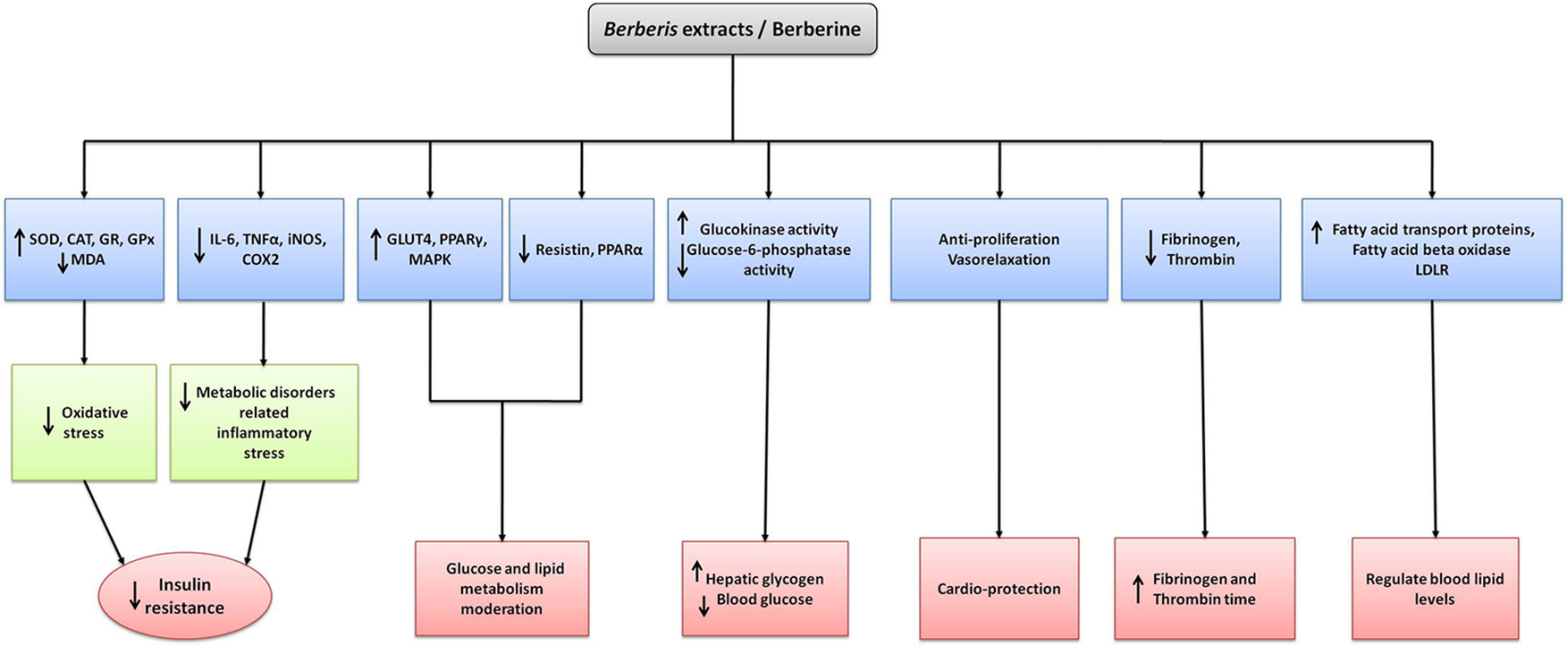
The mechanism of action of extracts and its major isolated alkaloid of *Berberis* species in the treatment of diabetes and metabolic syndrome. *Berberis* spp. and berberine upregulate the anti-oxidant enzymes while decreasing reactive oxygen species and inflammatory mediators which in turn decreases oxidative and inflammatory stresses and thus decreasing insulin resistance. Upstream regulating expression of GLUT4, PPARγ, MAPK and downstream regulation of resistin, PPARα results glucose and lipid metabolism moderation. Increase in AMPK and glucokinase activities while decrease in glucose-6-phosphate activity results in decreasing gluconeogenesis, restoring hepatic glycogen and blood glucose. Upregulating AMPK and p38 MAPK activities also cause increasing insulin action and decreasing lipid synthesis. Antiproliferative action and vasorelaxation results in cardioprotection whereas decrease in fibrinogen and thrombin results in increasing fibrinogen and thrombin time respectively. Increasing expression of fatty acid transport proteins, fatty acid beta oxidase and LDLR aids in regulating blood lipid levels.

Some of the mechanisms of *Berberis* species and BBR against diabetes and metabolic diseases are depicted in **Figure 4**.

## *In Vivo* Activities Against Diabetes and Metabolic Diseases

Extracts of *Berberis* species and their components, especially alkaloids, have been documented for their potential activity against T2DM and MS in *in vivo* models (**Table 3**). In the MS condition, BBR improved vascular inflammation and remodeling that was found to be correlated with the ability to inhibit p38 MAPK activation, ATF-2 phosphorylation, and MMP-2 expression (Li et al., 2015). Long-term treatment with BBR diminished the adipose tissue weight and decreased the renal injury (MS related diseases) in spontaneously hypertensive rats (Kishimoto et al., 2015). In normal diet-fed mice treated with BBR, hepatic CD36 expression and TG levels were increased; however, these effects were prevented when hepatic CD36 was silenced with an adenovirus containing CD36-specific short hairpin RNAs (shRNA) (Choi et al., 2017). BBR also improved the insulin-mediated vasodilatation of mesenteric arteries in diabetic rats through upregulation of insulin receptor-mediated signaling and increasing vascular InsS (Geng et al., 2016). Similarly, BBR increased both InsR and the low-density lipoprotein receptor (LDLR) expression, which resulted in a cellular response against InsR (Kong et al., 2009). In hyperlipidemic hamsters, the cholesterol-lowering effect of BBR was found to be due to its activity on upregulation of hepatic LDLR (Kong et al., 2004). Administration of BBR in hyperlipidemic and InsR rats decreased blood free fatty acid levels and increased the activity of lipoprotein lipase, leading to the amelioration of blood lipid and glucose metabolism (He et al., 2004). BBR administration resulted in the decrease of fasting blood glucose (FBL) level and ameliorated glycogen structural fragility (Li et al., 2019). Furthermore, BBR displayed beneficial effects in the treatment of obesity, and this was in part *via* improvement of adipose tissue fibrosis (Wang L. et al., 2018). BBR was reported to act in the liver to regulate lipid utilization and to maintain whole-body energy metabolism by mediating autophagy and FGF21 activation (Sun Y. et al., 2018). Additionally, BBR is also reported to reduce the systemic low-grade inflammation of T2DM mice to alleviate disease, and this effect may be achieved through regulating the gut microbes or inhibiting the TLR4 signaling pathway (Cao et al., 2017). Other *in vivo* investigations also showed the hypoglycemic effects of BBR through the improvement in gut-derived hormones and the attenuation of both intestinal mucosal mechanic and immune barrier damages (Gong et al., 2017). In the same way, the gut microbiota modulation was also suggested to be an effective mechanism of the antidiabetic effect of BBR (Han et al., 2011). The lipid-lowering effect of BBR chloride treatment in hyperlipidemic rats was found to be associated with a global change in the metabolism of lipids, carbohydrates, and amino acids as well as the structure of microbiota (Li et al., 2016).

**Table 3:** *In vivo* activity extracts and/or isolated compounds from *Berberis* species against diabetes and metabolic diseases.

Extracts from *Berberis* spp./isolated compounds	Model	Outcomes	References
Berberine			
Berbamine (BBA)	STZ-induced diabetic Sprague-Dawley rats	↑metabolic enzymes activities and preserved the glucose homeostasis	(Chandrasekaran et al., 2018)
Berberine (BBR)	Specific-pathogen-free male C57BL/6 mice	prolonged activation of AMPK BBR-induced ↑CD36 expression and fatty acid uptake	(Choi et al., 2017)
Berberine (BBR)	male Sprague–Dawley diabetic rats	↑DVIS and ↑mesenteric vasodilatation by insulin receptor-mediated signaling upregulation.	(Geng et al., 2016)
Berberine (BBR)	male Wistar rats	↓secretion of inflammatory factors and ↑vascular remodeling. Inhibition of p38 MAPK activation, ATF-2 phosphorylation, and MMP-2 expression.	(Li et al., 2015)
Berberine (BBR)	Male spontaneously hypertensive rats	↓BWG, ↓retroperitoneal adipose tissues, ↓mesenteric adipose tissues, and ↓urinary albumin excretion.	(Kishimoto et al., 2015)
Berberine (BBR)	T2DM STZ-induced Wistar rats	↓FBGL, ↓FSIL, ↑InsS, ↑InsR-mRNA, and ↑PKC activity in the liver.	(Kong et al., 2009)
Berberine (BBR)	hyperlipidemic hamsters	↓TC, ↓LDL-C, ↑hepatic LDLR mRNA, and ↑hepatic LDLR protein	(Kong et al., 2004)
Berberine (BBR)	Hyperlipidemic and IR rats	↓TC, ↓TG, ↓ApoB, ↓LDL-C, ↓FFA, ↑HDL-C, ↑ISI, ↑ApoAI, and ↑lipoprotein lipase activity	(He et al., 2004)
Berberine (BBR)	T2DM db/db mice	↓FBGL and ameliorated glycogen structural fragility	(Li et al., 2019)
Berberine (BBR)	HFD Obese rats	↓BWG, ↑glucose tolerance, ↓collagen deposition and reversed the upregulation of fibrosis related genes in the adipose tissue of HFD.	(Wang L, et al., 2018)
Berberine (BBR)	Liver-specific SIRT1 knockout mice	Regulation of lipid usage and preserved whole-body energy metabolism *via* autophagy and FGF21 activation.	(Sun Y, et al., 2018)
Berberine (BBR)	Rat islets	Inhibition of glucose-stimulated insulin secretion with AMPK activation, ↓OCR and ↓ATP production induced by high glucose, and attenuation of glucose-stimulated expression of fatty acid synthase	(Bai et al., 2018)
Berberine (BBR)	T2DM mice	↓systemic low-grade inflammation to alleviate disease, by regulating the gut microbes and/or inhibiting TLR4 signaling pathways.	(Cao et al., 2017)
Berberine (BBR)	Diabetic rats	hypoglycemic effects associated to ↑ gut-derived hormones.	(Gong et al., 2017)
Berberine (BBR)	T2DM rats	↓MALA, ↑InsR and ↑liver enzymes by	(Almani et al., 2017)
Berberine (BBR)	Diabetic rats	Attenuation of hyperglycemia, oxidative stress and inflammation by potentiation of the antioxidant defenses and up-regulation of PPARγ expression	(Mahmoud et al., 2017)
Berberine (BBR)	SD rats	↓2h-PPG level by local inhibition of intestinal DPP-IV.	(Wang J, et al., 2016)
Berberine (BBR)	Diabetic rat model	↓ expressions of Nrf2 and HO-1	(Tao et al., 2017)
Berberine (BBR)	Diabetic rats	Inhibition of hepatic gluconeogenesis *via* the regulation of the LKB1-AMPK-TORC2 signaling pathway.	(Jiang et al., 2015)
Berberine (BBR)	Diabetic hamsters	↓BGL, ↓TC, ↓TG, ↓FFA, ↓LDL-C, ↓Glucose, ↓insulin levels, ↓malondialdehyde, ↓thiobarbituric acid-reactive substance, and ↓8-isoprostane levels, ↑expression of skeletal muscle glucose transporter 4 mRNA and ↓liver LDL receptor mRNA expression.	(Liu et al., 2015)
Berberine (BBR)	Zucker Diabetic Fatty Rats	↓HbA1c, ↓TC, ↓TG, ↑insulin secretion, regulation of glucose and lipid metabolism and activation of pAMPK.	(Dong et al., 2016)
Berberine (BBR)	db/db mice and high-fat–fed Wistar rats	↓BWG, ↑glucose tolerance, ↓TG, and ↑ insulin action	(Lee et al., 2006)
Berberine (BBR)	Diabetic rats	Direct inhibition of liver gluconeogenesis	(Xia et al., 2011)
Berberine (BBR)	Diabetic rats	Intestinal microbiome modulation	(Han et al., 2011)
Berberine (BBR)	Diabetic rats	Lipid metabolism regulation and ↑ elimination of free radicals	(Tang et al., 2006)
Berberine (BBR)	Diabetic rats	PPAR α/δ up-regulation and PPARδ repression in liver	(Zhou et al., 2008)
Berberine (BBR)	Non-obese Diabetic rats	Regulation of MAPK activity to control the differentiation of Th17 and Th1	(Cui et al., 2009)
Berberine (BBR)	Diabetic rats	Promotes secretion of glucagon-like peptide type I	(Lu et al., 2009)
Berberine (BBR)	Diabetic rats	Tyrosine phosphatase 1B activity inhibition and insulin-like effect	(Chen et al., 2010)
Berberine (BBR)	Diabetic hamster	Up-regulation of LXRα, PPARα, and down-regulation of SREBPs	(Liu et al., 2010)
Berberine (BBR)	Diabetic rats	↓ intestinal disaccharidases and β-glucuronidases activities	(Liu et al., 2008)
Berberine (BBR)	Diabetic rats	Glucose metabolism modulation by GnRH-GLP-1 and MAPK pathway in the gut	(Zhang et al., 2014)
Berberine chloride (BC)	Diabetic rats	↓FBG, ↓WBC, ↓HbAlc ↑plasma insulin, ↑hemoglobin, ↑RBC, ↑Ht, ↑MCH and ↑MCHC.	(Chandirasegaran et al., 2017)
Berberine chloride (BC)	Diabetic rats	↓TC, ↓TG, ↓phospholipids, ↓LDL-C, ↓VLDL, ↓LOOH, ↓TBARS. ↑SOD, ↑CAT, ↑GPx, non-enzymatic antioxidant (↑GSH, ↑vitamin C, ↑vitamin E) and ↑IRS-1, ↑PKB, ↑Akt and ↑GLUT-4)	(Chandirasegaran et al., 2019)
Berberine fumarate (BF)	T2DM rats	↑metabolic disorder and ↓ inflammation by ↓over-expression of TLR4 and p-JNK and ↑PI3K and VGLUT2 expression.	(Cui et al., 2018)
**Combination of berberine and other compounds/extracts**			
Berberine chloride (BC), oryzanol and vitamin B2	Male Wistar hyperlipidemic rats	↓lipid effect without apparent adverse side effects.	(Li et al., 2016)
Berberine (BBR), *Ortosiphon staminensis*, policosanol, red yeast rice extract, folic acid and coenzyme Q10	Rats	↓TC, ↓LDL-C, ↓DBP, ↓TG, and ↑HDL-C. antihypertensive effect, which allows an effective control of blood pressure	(Rozza et al., 2009)
Berberine - Metformin Hybrid (BMH473)	T2DM obese rats	↑maintaining glucose and ↑ lipid homeostasis, ↑antihyperlipidemic activity.	(Jia et al., 2019)
berberine (BBR) and Timosaponin B2 (TB-2)	Goto-Kakizaki rats	↑anti-diabetic efficacy.	(Huang et al., 2019)
berberine (BBR) and Glycyrrhizic acid	Rats	↓FBG, and ↑Insulin level	(Qiao et al., 2018)
Berberine (BBR) with resveratrol	High fat diet-induced mice	↓TC, ↓TG, and ↓LDL-C	(Zhu et al., 2018)
Berberine (BBR) and Gelucire44/14	diabetic mice	Gelucire44/14 showed potential ↑oral absorption of BBR thus ↑ anti-diabetic efficacy.	(Sun J, et al., 2018)
Berberine organic acid salts (BOAs), including berberine citrate, berberine fumarate, berberine malate, and berberine succinate	T2DM rats	↑ hypoglycemic effects	(Li et al., 2017)
Berberine (BBR) and *Coptis chinensis* extract (CCE)	T2DM rats	↑pancreatic insulin secretion *via* ↑ islet β-cell proliferation and ↑ protein expression of PARP-1.	(Jiang et al., 2017)
Berberine (BBR) combined with Canagliflozin	Diabetic mice	↓FBG and ↓insulin. Antidiabetic effect associated with ↑ pAMPK and ↓ TNFα in kidneys.	(Cai-Ming et al., 2016)
Berberine (BBR) and Ginsenoside Rb1 (Rb1)	Diabetic mice	Improved abnormal metabolism of glucose and lipid.	(Shang et al., 2015)
Berberin glycyrrhizinate complex salt (BGC)	GK rats	↓PBG, ↓insulin level, ↓GSP, ↓LDL-C and ↓MDA, and ↑ histopathological changes in kidney and pancreas.	(Wang et al., 2014)
***Berberis* extracts**			
*B. aristata* roots (ethanolic extract)	Diabetic rats	↓dose-dependent in hyperglycemia, ↓TC, ↓TG, ↓AST, and ↓ALT levels of serum, ↓serum creatinine and ↓blood urea.	(Mittal et al., 2012)
*B. aristata* stem (ethanolic extract)	T1DM and T2DM albino rats	↑Liver glycogen and ↓FBS	(Rameshwar et al., 2009)
*B. aristata* roots (ethanolic extract)	STZ-induced diabetic rats	↓PBG	(Pareek and Suthar, 2010)
*B. aristata* stem bark (aqueous extract)	STZ-induced diabetic rats	↓TC and ↑HDL-C	(Ahamad et al., 2012)
*B. aristata* bark (ethanolic extract)	alloxan-induced diabetic rats	↓PBG	(Semwal et al., 2008)
*B. aristata* stem bark (methanolic extract)	Alloxan-Induced DiabeticRats	↓PBG	(Gupta et al., 2010)
*B. aristata* roots (methanolic-water extract	Diabetic rabbits	↓PBG	(Akhtar et al., 2008)
*B. aristata* roots (water-ethanolic extract)	Diabetic rats	Regulated glucose homeostasis *via* ↓ gluconeogenesis and ↓oxidative stress.	(Singh and Kakkar, 2009)
*B asiatica* roots (water-ethanolic extract)	Diabetic rats	↓BW	(Singh and Jain, 2010)
*B. dictyophylla* roots (extract)	Diabetic mice and normal mice	↓FBG, ↓ICAM-1, ↓ANGII, and ↓SOD in serum expression	(Yue et al., 2013)
*B. holstii* roots (aqueous extract)	Alloxan-induced diabetic male mice	↓FBGL	(Kimani et al., 2017)
*B. integerrima* roots (aqueous extract)	Diabetic male Wistar rats	↑renal by control of blood glucose and renal protective effects.	(Ashraf et al., 2013)
*B. integerrima* fruits (anthocyanin fraction)	Diabetic Male Sprague Dawley rats	↓FBG, ↑ liver glycogen level, and ↑ body weight.	(Sabahi et al., 2016)
*B. julianae* roots (methanolic extract)	T2DM mice	↑ GLUT4 translocation, ↑ oral glucose tolerance, ↑LDL-C, ↓BWG, ↓blood glucose and ↓other related blood-lipid contents.	(Yang et al., 2014)
*B. lycium* roots (aqueous extract)	Diabetic rabbits	↓ FBG.	(Ahmad and Alamgeer, 2009)
*B. lycium* extract (BLE)	Diabetic rabbits	↓TG, ↓TC, ↓LDL-C, and ↑HDL-C	(Ahmad et al., 2008)
*B. lycium* leaves (methanolic extract)	Female diabetic rabbits	↓FBG	(Hussain et al., 2017)
*B. lycium* roots (ethanolic extract)	Alloxan treated rats	↓FBG	(Gulfraz et al., 2007)
*B. lycium* roots (powder)	Broilers chickens	↓TG, ↓TC, ↓LDL-C, and ↑HDL-C	(Chand et al., 2007)
*B. lycium* roots (aqueous extract)	Diabetic rats	↓FBG, ↓TC, ↓TG, ↓LDL-C, ↓VLDL, ↓SGOT, ↓SGPT, and ↓ALP	(Mustafa et al., 2011)
*B. lycium* fruits (aqueous extract)	Diabetic rats	↓TC, ↓TG, ↓LDL-C, ↓VLDL, and ↓MDA	(Rahimi Madiseh et al., 2014)
*B. lycium* root (methanolic extract) and berberine (BBR)	Diabetic rats	↓FBG, ↑glucose tolerance, positive serum lipid profiles, glycosylated hemoglobin and body weight.	(Gulfraz et al., 2008)
*B. vulgaris* roots (aqueous extract)	Diabetic rats	↓TC and ↓TG.	(Meliani et al., 2011)
*B. vulgaris* fruits (aqueous and hydro-ethanolic extract)	T1DM Rats	↑ serum glucose levels, ↑ serum alanine aminotransferase activities, and ↓ HbA1c.	(Karami et al., 2016)
*B. vulgaris* fruits (ethanolic extract)	Diabetic rats	↑total antioxidant levels, ↓MDA and ↓FBG, and ↑mRNA level of GK	(Hemmati et al., 2016)
*B. vulgaris* fruits (Hydro-ethanolic extract)	Diabetic rats	↓ liver damage by influencing hepatic histopathological and biochemical markers	(Rahimi-Madiseh et al., 2017)
Jatrorrihizine	Hyperglycemic mice	↓FBG and ↑aerobic glycolysis	(Yan et al., 2005)
Jatrorrihizine and berberine	Diabetic rats	↓FBG. Berberine > Jatrorrihizine	(Fu et al., 2005)
Palmatine	Normal rats	↓FBG.	(Patel and Mishra, 2011)

The ↑ and ↓ signs show significant increase and significant decrease, respectively, of evaluated factors during mentioned studies.

On the other hand, BBR protects against metformin-associated lactic acidosis (MALA) in streptozotocin (STZ)-induced T2DM (Almani et al., 2017). BBR attenuated hyperglycemia and its associated oxidative stress and inflammation through, possibly, the potentiation of the antioxidant defenses and upregulation of PPARγ expression (Mahmoud et al., 2017). BBR decreased 2-hour postprandial plasma glucose (2h-PPG) level in STZ-induced diabetic rats by locally inhibiting intestinal DPP-IV (Wang J. et al., 2016). Moreover, BBR also reduced the blood glucose level in diabetic rats, improving the blood lipid and decreasing the retinal vascular injury, suggesting its association with the reduced expressions of Nrf2/HO-1 (Tao et al., 2017). BBR also upregulated protein expressions of LKB1, AMPK, p-AMPK, and p-TORC2 and also inhibited the translocation of TOCR2 into the cell nucleus (Jiang et al., 2015). Moreover, BBR was also found to be effective in lowering blood glucose and lipid levels, reducing the body weight, and alleviating the oxidative stress in diabetic hamsters (Liu et al., 2015).

The anti-diabetic effect of BBR was suggested to be mainly due to its activity in the regulation of glycometabolism and lipometabolism and the activation of AMPK (Lee et al., 2006; Dong et al., 2016). BBR improved glucose metabolism through an insulin-independent pathway (Xia et al., 2011). BBR also significantly inhibited the progression of diabetes induced by alloxan, and the effect of BBR on diabetes was suggested to be associated with its hypoglycemic effect, modulating lipids metabolic effects and its ability to scavenge free radicals (Tang et al., 2006). BBR improved glucolipid metabolism in diabetic rats both in the blood and liver, possibly through modulating the metabolic related PPARα/δ/γ protein expression in liver (Zhou et al., 2008). BBR targeted MAPK to suppress Th17 and Th1 differentiation in T1DM NOD mice and showed a novel role of ERK in Th17 differentiation through downregulation of STAT3 phosphorylation and RORt expression (Cui et al., 2009). Altered hepatic SREBPs, LXRα, and PPARα transcriptional programs were suggested to be involved in the therapeutic mechanisms of BBR on fat-induced hepatic insulin resistance (FIHIR) in T2DM hamsters (Liu et al., 2010). The inhibitory effect on intestinal disaccharidases and *β*-glucuronidase of BBR might be one of the mechanisms for BBR as an antihyperglycaemic agent (Liu et al., 2008). BBR caused the glucose metabolism modulation by the GnRH-GLP-1 and MAPK pathway in the gut (Zhang et al., 2014). The treatment of BBR chloride notably protected the blood components (Chandirasegaran et al., 2017) and significantly reversed the abnormal levels of lipids, oxidant status, and insulin signaling molecules in the diabetic rat model (Chandirasegaran et al., 2019). BBR also reduced the release of lipopolysaccharides and ameliorated inflammation by reducing the level of lipolysaccharide binding protein (LBP), thus alleviating intestinal injury and improving InsR (Cui et al., 2018).

The combination of *Ortosiphon staminensis*, policosanol, red yeast rice extract, BBR, folic acid, and coenzyme Q_10_ provided an antihypertensive effect, which allowed for an effective control of blood pressure in patients with MS (Rozza et al., 2009). The berberine-metformin hybrid compound BMH473 was found to be beneficial for maintaining glucose and lipid homeostasis in T2DM rats, and it exhibited better anthyperlipidaemic effects compared to metformin and BBR alone (Jia et al., 2019).

Combining timosaponin B2 (TB-2) and BBR in a single formulation enhanced the anti-diabetic efficacy by improving the intestinal absorption (Huang et al., 2019). Glycyrrhizic acid was also reported to improve the oral absorption of BBR by inhibiting P-gp, and it thus increased the anti-diabetic effects of BBR in db/db mice (Qiao et al., 2018). Lipid-lowering effects of BBR were also reported to be increased with resveratrol, which may be associated with upregulation of a low-density-lipoprotein (LDL) receptor (Zhu et al., 2018). Similarly, gelucire44/14 was found to enhance the oral absorption of BBR and thus improve the antidiabetic efficacy of BBR (Sun J. et al., 2018). Berberine organic acids (BOAs) were found to be comparable to berberine hydrochloride (BH) in terms of hypoglycaemic effects, they were but superior with regard to safety from hyperchloraemia in T2DM rats (Li et al., 2017). *Coptis chinensis* (containing berberine) and BBR exerted similar effects when used for the treatment of T2MD rats, mainly *via* the stimulation of the pancreatic secretion of insulin (Jiang et al., 2017). Berberine chloride was a stronger antidiabetic agent than BBR or canagliflozin alone with fewer side effects on kidneys in the diabetic mice (Cai-Ming et al., 2016). BBR and ginsenoside Rb1 (Rb1) improve abnormal metabolism of glucose and lipid (Shang et al., 2015).

Extracts of *Berberis* plants have shown interesting results in *in vivo* models. The ethanolic extract of *B. aristata* showed antidiabetic activity due to its significant dose-dependent reduction effect on the blood glucose levels (Semwal et al., 2008; Mittal et al., 2012), which were also reported to be better than glibenclamide (Rameshwar et al., 2009) and comparable to metformin in diabetic rats (Pareek and Suthar, 2010). In addition, the aqueous extract of *B. aristata* showed significant antidiabetic activity, decreased total cholesterol, increased HDL-C levels, and prevented the body weight loss in diabetic rats (Ahamad et al., 2012).

The aqueous extract of *B. lycium* roots showed an antihyperlipidemic effect (Ahmad et al., 2008). *B. lycium* leaf extracts alleviated lipid profile levels and might be used efficiently in hyperglycemic and diabetic patients (Hussain et al., 2017). Also, the root extract of *B. lycium* reduced the serum glucose levels in normal and diabetic rats (Gulfraz et al., 2007). In chicken Broilers, the powder of *B. lycium* reduced the serum cholesterol (Chand et al., 2007). The oral administration of extracts of *B. lycium* showed hypoglycemic activity (Mustafa et al., 2011) and alleviated lipid profile levels (Rahimi Madiseh et al., 2014). Similarly, the methanolic extract of the *B. lycium* root and its main alkaloid BBR showed hypoglycemic activity (Gulfraz et al., 2008) and showed antiglycation activity (Khan et al., 2014).

On the other hand, in diabetic rats, the beneficial effects of *B. vulgaris* extracts showed positive effects in attenuating the side effects of T2DM (Karami et al., 2016), ameliorating oxidative stress (Hemmati et al., 2016), decreasing the liver damage by influencing hepatic histopathological and biochemical markers (Rahimi-Madiseh et al., 2017), and showed that the serum cholesterol and serum triglycerides levels were decreased (Meliani et al., 2011).

Other species of *Berberis* have also been studied. For instance, *B. asiatica* hydro-ethanolic root extracts have shown to be a potent orally effective antidiabetic extract (Singh and Jain, 2010). Likewise, the *B. dictyophylla* cortex could significantly reduce the level of fasting blood glucose, ICAM-1, and ANG II expression (Yue et al., 2013). The *B. holstii* extract showed the reduction of blood glucose levels (Kimani et al., 2017). Furthermore, the aqueous extract of *B. integerrima* roots improved renal dysfunction in STZ-induced diabetic rats through controlling blood glucose, and it also showed renal protective effects (Ashraf et al., 2013). The anthocyanin fraction of the fruits of *B. integerrima* also showed hypoglycemic effects (Sabahi et al., 2016). Moreover, the methanolic extract of *B. julianae* roots was also reported to possess promising beneficial effects for the treatment of T2DM with the possible mechanism *via* stimulating AMPK activity (Yang et al., 2014).

Other alkaloids isolated from *Berberis* species have also shown promising activities against T2DM and MS. For example, berbamine increased the activity of metabolic enzymes and preserved the glucose homeostasis in HFD/STZ induced diabetic rats (Chandrasekaran et al., 2018). Jatrorrihizine (JAT) induced an important decrease in FBG in normal and hyperglycemic mice, attributed to improve in aerobic glycolysis (Yan et al., 2005). JAT, BBR, and a combination of BBR and JAT decreased the FBG of diabetic and normal mice at different degrees. JAT also possessed the function of decreasing FBG, which was found less than that of BBR at the same dose level (Fu et al., 2005). Palmatine was also found to decrease FBG and suppressed the increase of blood glucose level in normal rats (Patel and Mishra, 2011).

## Studies in Humans

Several pilot studies as well as pre-clinical studies and clinical trials have evaluated the beneficial effects of *Berberis* extracts and isolated compounds on diabetes, metabolic syndrome, and other metabolic diseases (**Table 4**).

**Table 4:** Studies in diabetic and/or metabolic syndrome patients using treatment with extract and/or isolated compounds of *Berberis* species.

*Berberis* spp./isolated compound	Study design/Model	Results	References
Berberine			
Berberine (BBR, 0.05g, 4 tablets/time, 3 times/day)	MS patients (*n*=80) RCT, 1 month	↓FBG, ↓PBG, ↓InsR, ↓TG, ↓TC, ↓hs-CRP, and ↓IL-6 and ↓TNF-α	(Cao and Su, 2019)
Berberine (BBR, 0.5 g, 2 times/day)	T2DM patients (*n* = 300), double-blind, RCT, 16 weeks	↓FPG	(Ming et al., 2018)
Berberine (BBR, 0.5 g, 3 times/day)	MS patients (*n*=24) double-blind, placebo-controlled, RCT, 3 months	↓WC, ↓SBP, ↓TG, ↓AUC of glucose, ↓AUC of insulin, ↓insulinogenic index, and ↑Matsuda index	(Pérez-Rubio et al., 2013)
Berberine (BBR, 0.4 g, 3 times/day)	T2DM patients (*n*=114), RCT, 6 months	↓HbA1c, ↓BUN, ↓SP, ↓hs-CRP, ↓ESR, and ↓eGFR	(Li et al., 2018)
Berberine (BBR, 0.5 g, 2 times/day)	Mild mixed hyperlipidemia (*n*=32), double-blind, RCT, 12 weeks	↓TC, ↓LDL-C and ↓TG.	(Kong et al., 2004)
Berberine (BBR, 1 g, 1 time/day)	T2DM and mixed hyperlipidemia patients (*n*=116), double-blind, RCT, 3 months	↓FPG, ↓PPG, ↓HbA1c, ↓TG, ↓TC, ↓LDL-C, and ↑GDR	(Zhang et al., 2008)
Berberine (BBR, 0.5 g, 3 times/day)	Newly diagnosed T2DM patients (*n*=36) double-blind, RCT, 3 months	↓HbA1c, ↓FBG, ↓PBG, ↓TG, ↓TC ↓FPI, ↓IR, and ↓LDL-C.	(Yin et al., 2008)
Berberine (BBR, 0.5 g, 2 times/day)	Hyperlipidemic patients (*n* =86), Open study, 3 months	↓LDL-C, ↓TC and ↓TG.	(Zhao et al., 2008)
Berberine (BBR, 0.3g, 3 times/day)	MS patients (*n*=41) Double‐blind, RCT, 3 months	↓BMI, and ↓leptin levels, ↓leptin/adiponectin ratio, ↓HOMA-IR, and ↑IS	(Yang et al., 2012)
Berberine (BBR, 0.5 g, 3 times/day)	PCOS and IR patients (*n*=89) randomized, single center, placebo-controlled, 3 months	↓WHR, ↓TC, ↓TG, ↓LDLC, ↓FPG, ↓HOMA-IR, ↓AUC of insulin, ↑HDLC, and ↑SHBG	(Wei et al., 2012)
Berberine (BBR, 1.0 g, 1 time/day)	T2DM and dyslipidemic patients (*n* = 116) double-blind, placebo-controlled and multiple-center trial consisting of a screening visit, RCT, 2-week	↓FFA	(Gu et al., 2010)
Berberine (BBR, 1.0 g, 1 time/day)	T2DM patients with fasting blood glucose (*n* = 96), 2 months	↓FBG, ↓HA1c, ↓TG, and ↓insulin levels	(Zhang et al., 2010)
Berberine (BBR, 0.5 g, 2 times/day)	T2DM patients (*n*=228) double-blind randomized controlled placebo, 4 weeks	↓FPG, ↓PMBG, and ↓FA.	(Rashidi et al., 2018)
Berberine (BBR, 0.5 g, 2 times/day)	T2DM patients (*n*=30), open labelled, observational and single centre study, 12 weeks	↓FBG, ↓PPBG, and ↓GHb	(Dange et al., 2016)
Berberine (BBR, 0.3 g, 3 times/day)	T2DM patients (*n*=30), 8 weeks	↓BMI, ↓FBG, ↓HbAlc, ↓fasting insulin, ↓TG, ↓TC, ↓HDL-C, ↓LDL-C, ↓CPR, ↓TNF-α, and ↓LPS	(Chen et al., 2016)
Berberine (BBR, N.I., 2 times/day)	T2DM patients (*n*=41), open-label interventional RCT, 3 months	↓HbA1C, ↓FBG, and ↓PPG	(Rao, 2017)
Berberine (BBR, 0.3 g, 3 times/day)	Mild hyperlipemic patients (*n*=97) Double‐blind, RCT, 3 months	↓TG, ↓TC, and ↓LDL-C	(Wang L et al., 2016)
Berberine (BBR, 0.4 g, 1 time/day)	Hypercholesterolemia in tolerance to more than one statin (*n*=91), 3 months	↓ LDL-C and ↓TG.	(Cicero and Ertek, 2008)
**Berberine combined with others compounds and extracts**			
Berberine (BBR, 1.0 g, 1 time/day.) and simvastatin (SIMVA)	Hypercholesterolemic patients (*n*=63), double-blind, RCT, 2 months	↓LDL-C, ↓TC, and ↓TG	(Kong et al., 2008)
(Berberine, BBR, 0.5 g; red yeast, 200 mg; and policosanol, 10 mg; 1 time/day)	Hypercholesterolemic patients (*n*=50), double-blind, single-centered, placebo-controlled, RCT, 6 weeks	↓TC, ↓LDL-C, ↓TG, ↑FMD, and ↑InsS	(Affuso et al., 2010)
(Berberine, BBR, 0.5 g; policosanols, 10 mg; and red yeast rice, 200 mg; 1 time/day)	Hypercholesterolemic patients (*n*=135) randomized, double-blind, EZE-controlled, 6 months	↓LDL-C, and ↓TG	(Pisciotta et al., 2012)
Armolipid Plus ™ composed by (Berberine, BBR, 0.5 g; red yeast rice, 200 mg; policosanol, 10 mg; folic acid, 0.2 mg; coenzyme Q_10_, 2.0 mg; and astaxanthin, 0.5 mg; 1 time/day)	Hypercholesterolemic patients (*n*=106), single-blind, single centered, placebo-controlled, RCT, 12 months	↓TC, ↓LDL-C, and ↓InsR	(Marazzi et al., 2011)
Armolipid Plus ™ composed by (Berberine, BBR, 0.50g; red yeast rice, 200 mg; policosanol, 10 mg; folic acid, 0.2 mg; coenzyme Q_10_, 2.0 mg; and astaxanthin, 0.5 mg; 1 time/day)	Hyperlipidemic patients (*n*=102), double-blind, parallel, controlled, Multiple centered, placebo-controlled, RCT, 12 weeks	↓LDL-C, ↓apo B-100, ↓TC/HDL-C, ↓ApoB/ApoA1 ratio, and ↑ApoA1	(Sola et al., 2014)
Armolipid Plus ™ composed by (Berberine, BBR, 0.5g; red yeast rice, 200 mg; policosanol, 10 mg; folic acid, 0.2 mg; coenzyme Q_10_, 2.0 mg; and astaxanthin, 0.5 mg; 1 time/day)	Dyslipidemic patients (*n* = 1751) Double‐blind, RCT, 16 weeks	↓TC and ↓LDL-C	(Trimarco et al., 2011)
Armolipid Plus ™ composed by (Berberine, BBR, 0.5g; red yeast rice, 200 mg; policosanol, 10 mg; folic acid, 0.2 mg; coenzyme Q_10_, 2.0 mg; and astaxanthin, 0.5 mg; 1 time/day)	Hypercholesterolemic patients (*n*=66), single-blind, placebo-controlled, RCT, 3 weeks	↓TC, ↓LDL-C, and ↓TG	(Gonnelli et al., 2015)
Armolipid Plus ™ composed by (Berberine, BBR, 0.5 g; red yeast rice, 200 mg; policosanol, 10 mg; folic acid, 0.2 mg; coenzyme Q_10_, 2.0 mg; and astaxanthin, 0.5 mg; 1 time/day)	Moderate dyslipidemic and MS patients (*n*=30), double-blind, centered, placebo-controlled, RCT,	↓TC, ↓LDL-C, ↓leptin-to-adiponectin ratio, and ↑HDL-C	(Ruscica et al., 2014)
Armolipid Plus ™ composed by (Berberine, BBR, 0.5 g; red yeast rice, 200 mg; policosanol, 10 mg; folic acid, 0.2 mg; coenzyme Q_10_, 2.0 mg; and astaxanthin, 0.5 mg; 1 time/day)	Dyslipidemic with ischemic heart disease treated patients (*n*=100), single-blind, EZE-controlled, RCT, 12 months	↓LDL-C, ↓TC, ↓TG, and ↑HDL-C	(Marazzi et al., 2015)
Berberine (BBR, 500mg) and Armolipid Plus ™ Composed by (Berberine, BBR, 0.5 g; red yeast extract, 200 mg; policosanol, 10 mg; folic acid, 200 mg; coenzyme Q_10_, 2 mg; and astaxanthin, 0.5 mg; 1 time/day)	Hyperlipidemic patients (*n*=40) single-blind, no placebo-controlled, 4 weeks	↓TC, ↓LDL-C, ↓ApoB, ↓TG, and ↑HDL-C	(Cicero et al., 2007)
Body Lipid ™ composed by (Berberine, BBR, 0.5 g; red yeast rice, 10 mg; coenzyme Q_10_, 2 mg; and hydroxytyrosol, 5 mg; 1 time/day)	Hypercholesterolemic patients (*n* = 158) Double‐blind, RCT, 4 weeks	↓TC and ↓LDL-C	(D'Addato et al., 2017)
Berberine (BBR, 0.2g; monacolin K, 3 mg; chitosan, 10 mg; and coenzyme Q_10_, 10 mg; 1 time/day)	Hypercholesterolemic patients (*n* =36) Double-blind phase II placebo-controlled study, 12 weeks	↓nHDL-C, ↓LDL-C and ↓apoB	(Spigoni et al., 2017)
Estromineral lipid ™ composed by (Berberine, BBR, 0.5 g; soy isoflavones, 60 mg; *Lactobacillus sporogenes*, 1x10^9^ spores; calcium phosphate dehydrate, 137 mg; vitamin D_3_, 5 μg; and folic acid, 0.2 mg; 1 time/day)	Menopausal women (*n*=120) RCT, 12 weeks	↓TC, ↓LDL-C, and ↓TG	(Cianci et al., 2012)
Berberine (BBR, 1.0 g; phytosterols, 4 g; antioxidants, 2 capsules; probiotics, 12 billion colony forming units; fish oil, 2g; and soy, pea, and whey proteins, 40 g, 2-3 times/day)	CMS patients (*n*=44) open-label, 2-arm, RCT, 13 weeks	↓body mass, ↓fat mass, ↓TC, ↓LDL-C, ↓TG, ↓TC/HDL-C, ↓TG/HDL-C, ↓apoB/apoA1, and ↓hs-CRP.	(Dahlberg et al., 2017)
Berberine sulfate trihydrate (0.1 g, equiv. 69 mg berberine, BBR); Hop rho iso-alpha acids, 200 mg; vitamin D_3_, 500 IU; and vitamin K_1_ 500 μg; 2 times/day)	MS postmenopausal women patients (*n*=51), randomized, single-blind, 2-arm placebo-controlled, RCT, 14 weeks	↓serum OC, serum ↑25(OH)D, and ↑IGF-I	(Lamb et al., 2011)
Berberine (BBR, 0.5 g, 3 times/day) and methylglyoxal (0.5 g ×3 times/day)	T2DM patient (*n*=200), case–control study, 3 months	↓HOMA-IR, and ↓MGO	(Memon et al., 2018)
Berberine (BBR, 0.5 g; orthosiphon, 300 mg; red yeast rice, 60 mg; monacolin, 3 mg; policosanol, 10 mg; folic acid, 0.2 mg; and coenzyme Q_10_, 15mg; 1 time/day)	MS patients (*n*=1161), Double-blind, Randomized, controlled, 1 year	↓TC, ↓LDL-C, ↓HDL-C, ↓TG, ↓SBP, and ↓DBP	(Manzato and Benvenuti, 2014)
***Berberis* extracts**			
*B. aristata* stem powder (1.5 and 3 g in two divided doses daily)	T2DM with dyslipidemic patients (*n*=90) open parallel, RCT, 9 months	↓FBS, ↑HDL, ↓TC, ↓TG, and ↓LDL.	(Sharma et al., 2017)
Berberol ^®^ compose by *B. aristata* (Berberine, BBR, 1.0 g) and *S. marianum* (silymarin, 210 mg) and only *B. aristata* extract (Berberine, BBR, 1.0 g) 2 time/day	T2DM patients (*n*=69), single-blind, RCT, 120 days	↓IFG, ↓HbA1c, ↓TC, ↓TG, ↓LDL (only Berberol ^®^), ↓AST, and ↓ALT	(Di Pierro et al., 2013)
Berberol ^®^ compose by *B. aristata* (Berberine, BBR, 1.0 g) and *S. marianum* (silymarin, 210 mg) 2 times/day	T1DM patients (*n*=85) double-blind, randomized, placebo-controlled, 6 months	↓TIC, ↓HgbA1c, ↓FPG, ↓PPG, ↓TC, ↓TG, ↓LDL-C, and ↑HDL-C	(Derosa et al., 2016)
Berberol ^®^ compose by *B. aristata* (Berberine, BBR, 1.0 g) and *S. marianum* (silymarin, 210 mg) 2 times/day	Dyslipidemic patients (*n*=105), Double‐blind, RCT, 3 months	↓TC, ↓LDL-C, ↓TG, ↑HDL-C, ↓FPI, and ↓HOMA-IR	(Derosa et al., 2013)
Berberol ^®^ compose by *B. aristata* (Berberine, BBR, 1.0 g) and *S. marianum* (silymarin, 210 mg) 2 times/day	T2DM and MS patients (*n*=50) double-blind placebo-controlled, 6 months	↓BMI, ↓HOMA-R, ↓TC, ↓WC, ↓HbA1c, and ↓TF%	(Guarino et al., 2015)
Berberol ^®^ compose by *B. aristata* (Berberine, BBR, 1.0 g) and *S. marianum* (silymarin, 210 mg) 2 times/day	T2DM and MS patients (*n*=136), placebo RCT, 52 weeks	↓TC, ↑HDL-C, ↓TG, ↓LDL-C, ↓HOMA-R, ↓WC, ↓TF(%), ↓VF(%), ↓UA, ↓HbA1c, ↓SBP, and ↓DBP	(Guarino et al., 2017)
Berberol ^®^ compose by *B. aristata* (Berberine, BBR, 1.0 g) and *S. marianum* (silymarin, 210 mg) 2 times/day	T2DM patients (*n* = 26), 6 months	↓HbA1c, ↓basal insulin, ↓TC, ↓LDL-C, ↓TG, ↓HOMA-R, ↓ ALT, and ↓AST	(Di Pierro et al., 2012)
Berberol ^®^ compose by *B. aristata* (Berberine, BBR, 1.0 g) and *S. marianum* (silymarin, 210 mg) 2 times/day	Dyslipidemic patients (*n* =175), double blind, placebo-controlled, RCT, 6 months	↓FPG, ↓IC, ↓HOMA, and ↓dosage of statin	(Derosa et al., 2015a)
Berberol ^®^ compose by *B. aristata* (Berberine, BBR, 1.0 g) and *S. marianum* (silymarin, 210 mg) 2 times/day	Euglycemic, dyslipidemic subjects (*n*=137) double-blind, RCT, placebo-controlled, 6-months	↓FPG, ↓IC, and ↓HOMA-index	(Derosa et al., 2015b)
Berberol ^®^ compose by *B. aristata* (Berberine, BBR, 1.0 g) and *S. marianum* (silymarin, 210 mg), Berberol ^®^ + statin, and Berberol ^®^ +ezetimibe; 2 times/day	T2DM and hypercholesterolemic patients (*n*=45), 6-months	↓TC, ↓LDL-C, ↓HDL-C (only Berberol ^®^), ↓FPG, and ↓HbA1c.	(Di Pierro et al., 2015)
Berberol ^®^ K compose by *B. aristata* (Berberine, BBR, 1.0 g) and *S. marianum* (silymarin, 210 mg) and Monakopure™-K20, 50 mg; 1 time/day	Dyslipidemic patients (*n*=226), non-blind non-randomized, 6 months	↓TC, ↓LDL-C, ↓TG, and ↓CPK.	(Di Pierro et al., 2018)
Berberol ^®^ K compose by *B. aristata* (Berberine, BBR, 1.0 g) and *S. marianum* (silymarin, 210 mg), and Monakopure™-K20, 50 mg; 1 time/day	Low cardiovascular risk patients (*n*=73), double-blind, placebo-controlled, RCT, 3 months	↑FPI, ↓HOMA, ↓TC, ↓TG, ↓LDL-C, and ↓hs-CRP	(Derosa et al., 2017)
Berberol ^®^ K compose by *B. aristata* (Berberine, BBR, 1.0 g) and *S. marianum* (silymarin, 210 mg), and Monakopure™-K20, 50 mg; 1 time/day	Diabetic and dyslipidemic patients (*n* = 59), 6 months	↓HbA1c, ↓TC, ↓LDL-C), and ↓TG	(Di Pierro et al., 2017)
*B. aristata* (83.3 mg), *Cyperus rotundus* (83.3 mg), *Cedrus deodara* (83.3 mg)*, Emblica officinalis* (83.3 mg), *Terminalia chebula* (83.3 mg) and *T. bellirica* (83.3 mg) 1-6 timea/day	T2DM patients (*n*=93) Pilot RCT, 24 weeks	↓PBG, ↓FBG, ↓TC, and ↓HbA1c.	(Awasthi et al., 2015)
*B. vulgaris* fruit (aqueous extract, 3 g/day)	T2DM patients (*n*=31) Double‐blind, RCT, 3 months	↓TG, ↓TC, ↓LDL-C, ↓apoB, ↓glucose, ↓insulin, and ↑TAC.	(Shidfar et al., 2012)
*B. vulgaris* fruit (600 mg/day)	MS patients (*n*=106) Double-blind, RCT, 6 weeks	↓PAB	(Mohammadi et al., 2014)
*B. vulgaris* juice (10 c.c. of processed extract/day)	MS patients (*n*=57) Double-blind, RCT, 8 weeks	↓LDL-C, ↓TC/HDL-C ratio, ↑HDL, ↑IC, and ↑IR.	(Ebrahimi-Mamaghani et al., 2009)
*B. vulgaris* fruit (ethanolic extract 1 mg, 3 times/day)	T2DM patients (*n*=30) Double-blind, RCT, 8 weeks	↓SGL, ↓FG, and ↓HbA1c	(Moazezi and Qujeq, 2014)
*B. vulgaris* juice (480 mL/day)	women diagnosed with BBD (*n* =85), 8 weeks	↓IC, ↓C-peptide, ↓HOMA-IR, ↓glucose/insulin ratio, and ↑HOMA-B.	(Asemani et al., 2018)
*B. vulgaris* fruit (600 mg/day)	(*n* = 106) Double-blind, RCT, 6 weeks	↓LDL-C, ↓TC, ↑HDL-C, ↓anti-HSPs 27, ↓anti-HSPs 60, and ↓hs-CRP	(Zilaee et al., 2014)

The ↑ and ↓ signs show significant increase and significant decrease, respectively, of evaluated factors during mentioned studies. N.I., not informed.

The administration of BBR in patients with MS was found to be effective in regulating the blood glucose and blood lipid levels, improving the InsR, and reducing the level of inflammatory responses in the body (Cao and Su, 2019). BBR also decreased the waist circumference, systolic blood pressure (SBP), triglycerides, and total insulin secretion along with an increase in InsS (Pérez-Rubio et al., 2013). BBR was suggested as a promising new hypolipidemic drug that acts through signaling pathways distinct from those of statins in the treatment of hyper mild mixed hyperlipidemia patients (Kong et al., 2004). Besides, BBR has been shown to have a good potential as a drug to control lipid metabolism alone or in combination with other drugs for hyperlipidemic hepatitis or liver cirrhosis patients (Zhao et al., 2008). Moreover, BBR improved the InsS by limiting fat storage and adjusting adipokine profile in human preadipocytes and MS patients (Yang et al., 2012), and attenuated some of the metabolic and hormonal derangements in women with polycystic ovary syndrome (PCOS (Wei et al., 2012). The administration of BBR was found to be effective in the regulation of blood glucose and blood lipid in T2DM patients (Ming et al., 2018) and in improving diabetic kidney disease by reducing UACR and serum Cys C (Li et al., 2018). On the other hand, BBR had also shown glucose-lowering activity with a mechanism different from metformin and rosiglitazone (Zhang et al., 2010). In pilot study, BBR demonstrated a potent oral hypoglycemic activity with positive effects on lipid metabolism (Yin et al., 2008). Also, the benefits of BBR in lowering blood glucose, lipids, body weight, and blood pressure have been confirmed in T2DM and MS patients (Zhang et al., 2008). BBR played an important role in the treatment T2DM through downregulating the higher levels of free fatty acids (Gu et al., 2010). In another study, BBR reduced the fasting plasma glucose, post-meal blood glucose, and fructosamine; however, no signification changes were found in lipid profiles, fasting insulin, HOMA-IR, and HOMA-β% in T2DM patients (Rashidi et al., 2018).

In addition, BBR improved the glycemic parameters comparable to metformin in T2DM patients (Dange et al., 2016). BBR significantly ameliorated T2DM *via* modulation of *Bifidobacterium* species, TNF-α, and LPS (Chen et al., 2016). BBR improved the blood lipid level in mild hyperlipidemia patients (Wang L, et al., 2016). Likewise, it reduced the plasma LDL-C and TG in mixed hyperlipidaemic subjects (Cicero and Ertek, 2008).

The combination of BBR and simvastatin (SIMVA) in hypercholesterolemic patients significantly improved LDL-receptor upregulation and LDL-cholesterol downregulation compared to monotherapies, and the combined effect also reduce the statins dosage (Kong et al., 2008). The administration of BBR along with red yeast and policosanol on a daily basis was found to be effective in reducing cholesterol levels and was associated with the enhancement of endothelial function and InsS (Affuso et al., 2010). The administration of this supplementation in patients with familial hypercholesterolemia heterozygotes on stable treatment with LDL-C-lowering validated that the supplement reduced the LDL-C superior to that obtained by doubling the dose of statins (Pisciotta et al., 2012).

Also, the dietary supplement Armolipid Plus™ composed of BBR, red yeast rice, policosanol, folic acid, coenzyme Q_10_, and astaxanthin showed significant reduction of cholesterolemia and positive plasma LDL-C levels in elderly (statin-intolerant) hypercholesterolemic patients (Marazzi et al., 2011). Moreover, it reduced LDL-C levels as well as total cholesterol/HDLc and ApoB/ApoA1 ratios, and it increased the Apo A1; tjos demonstrated the improvements in CVD risk indicators in patients with hypercholesterolemia (Sola et al., 2014) and amelioration of blood lipids and significant reduction of global CVD risk in dyslipidemic patients (Trimarco et al., 2011). In patients with low- to moderate-risk hypercholesterolemia, Armolipid Plus ™ in association with a hypolipidic diet significantly reduced the total cholesterol and LDL-C levels (Gonnelli et al., 2015). In addition, Armolipid Plus ™ improved the lipid profile similar to a low dose of a standard statin and also increased the HDL-C levels and improved the leptin-to-adiponectin ratio in patients with moderate dyslipidemia and MS (Ruscica et al., 2014). Armolipid Plus™ alone or in combination with ezetimibe enhanced the lipid profile in statin-intolerant patients with coronary heart disease (Marazzi et al., 2015). BBR and Armolipid Plus™ could be a useful alternative to correct dyslipidemias and to reduce CVD risk in subjects with moderate mixed dyslipidemias (Cicero et al., 2007).

Other food supplements containing BBR, including Body Lipid™, were suggested as an alternative to pharmacological treatment for patients with mild-to-moderate hypercholesterolemia (D'Addato et al., 2017). A new nutraceutical formulation containing BBR, monacolin K, chitosan, and coenzyme Q_10_ has proven effective in reducing non-HDL/LDL-C levels, representing an emergent therapeutic strategy in dyslipidemic patients (Spigoni et al., 2017). On the other hand, the combination of BBR and isoflavones was found to be effective in lowering CVD risk factors in menopausal women with moderate dyslipidaemia (Cianci et al., 2012).

Treatment with BBR and rho iso-alpha acids, vitamin D3, and vitamin K1 produced a more favorable bone biomarker profile, indicative of healthy bone metabolism in postmenopausal women with MS (Lamb et al., 2011). In a case–control study, BBR is more effective in decreasing the serum MGO levels and InsR through increasing the glycemic control in newly diagnosed T2DM patients (Memon et al., 2018). The intake of the natural formulation (containing BBR, orthosiphon, red yeast rice equivalent to monacolin, policosanol, folic acid, and coenzyme Q_10_) has evidenced the effective control of plasma lipids and keeps borderline high blood pressure within normal values compared with diet alone (Manzato and Benvenuti, 2014).

Stem powder of *B. aristata* was found to be effective in improving glycemic control and lipid profiles with no major adverse effects on T2DM patients (Sharma et al., 2017). The effect of *B. vulgaris* extract on T2DM and MS patients has been widely studied in humans. The intake of 3 g/d of *B. vulgaris* fruits aqueous extract for 3 months may have beneficial effects on lipoproteins, apoproteins, glycemic control, and TAC in T2DM patients (Shidfar et al., 2012). *B. vulgaris* juice reduced oxidative burden in patients with MS (Mohammadi et al., 2014). Other study showed the beneficial effects of processed *B. vulgaris* on certain atherosclerosis risk factors in T2DM patients (Ebrahimi-Mamaghani et al., 2009). *B. vulgaris* fruit extract showed beneficial metabolic effects in T2DM patients, improving the glucose catabolism *via* the glycolysis pathway, stimulating the insulin secretion or improving the insulin function, and later decreasing the glucose uptake (Moazezi and Qujeq, 2014). Another study demonstrated that the *B. vulgaris* juice evoked regulatory roles on HOMA-IR and improved HOMA-B with the metabolic controlling insulin-related indices in benign breast disease (Asemani et al., 2018). Also, *B. vulgaris* supplementation in patients with MS significantly diminished anti-HSPs 27 and 60 and hs-CRP levels and improved lipid profiles (Zilaee et al., 2014). It is reported that the Hsp60 protein is able to induce the production of anti-Hsp60 antibodies, which leads to the destruction of β-islet cells. In the same way, Hsp60 acts as a proinflammatory signaling molecule, which plays a role in the non-resolved vascular inflammation, and this is recognized as one of the characteristic of T2DM (Juwono and Martinus, 2016). Others natural formulations containing *Berberis* have also been tested in humans. A clinical trial demonstrated that daily intake of polyherbal capsule composed by *B. aristata* and *Cyperus rotundus*, *Cedrus deodara*, *Emblica officinalis*, *Terminalia chebula*, and *T. bellirica* decreased the glucose level, enhanced lipid homeostasis, and maintained other serum biochemical levels to the normal in patients with T2DM (Awasthi et al., 2015).

The nutraceutical product Berberol^®^, containing a *B. aristata* extract (titrated in 85% BBR) plus a *Silybum marianum* extract (titrated in 60% silymarin), has been evaluated for its antidiabetic potential in humans. Berberol^®^ was demonstrated to be more effective than BBR alone (administered at the same dose), reducing HbA1c in T2DM patients (Di Pierro et al., 2013). The incorporation of Berberol^®^ into insulin therapy in patients with T1DM has the effect of a diminution of the insulin dose necessary for adequate glycemic control (Derosa et al., 2016). In dyslipidemic patients, Berberol^®^ has proven to be safe and effective in improving lipid profile, InsR, and adipocytokines levels (Derosa et al., 2013). Berberol^®^ also improved the cholesterol-lowering properties of statins and showed the positive effects on liver enzymes and glycemic control in patients with T2DM (Guarino et al., 2015). In addition, Berberol^®^ significantly lowered abdominal adiposity and decreased the circulating uric acid level in overweight/obese patients with T2DM (Guarino et al., 2017). Berberol^®^ was suggested as a good candidate for an adjunctive treatment option in diabetes, especially in patients with suboptimal glycemic control (Di Pierro et al., 2012). Berberol^®^ administered as a single or add-on therapy in statin-intolerant subjects is an effective treatment to improve the lipidic and glycemic profiles in T2DM and hypercholesterolemia patients (Di Pierro et al., 2015). The combination of Berberol^®^ and a reduced dosage of statin is found effective for the treatment of hyperlipidemia in patients intolerant to statins at high dosage (Derosa et al., 2015a) and in dyslipidemic euglycemic patients (Derosa et al., 2015b)

Berberol K^®^, was found to be a potentially good alternative in primary intervention in low cardiovascular-risk subjects with dyslipidemia, as an add-on therapy in mildly statin-intolerant patients, and as an alternative for dyslipidemic patients with a negative perception of statins (Di Pierro et al., 2017). Berberol K^®^ reduced lipid profile effectively and improved the inflammatory parameters under a safe dose (Derosa et al., 2017). It was also found to be effective in diabetic subjects with dyslipidemia statin intolerant or with diarrhea caused by IBS or metformin (Di Pierro et al., 2018).

Few studies have also reported the effectiveness of BBR in non-alcoholic fatty liver disease (NAFLD). NAFLD is a result of abnormal fat accumulation in the liver due to the reasons other than alcohol, and it is considered to be a hepatic manifestation of MS. NAFLD results in the overproduction of sugars and triglycerides and plays a central role in the development of InsR and various other glucose- and lipid metabolism-related diseases (Yki-Järvinen, 2014). Recently, Yan et al. (2015) conducted a randomized, parallel controlled, open-label clinical trial in 188 NAFLD patients. Patients received lifestyle intervention (LSI) or LSI and 15 mg of pioglitazone qd or LSI and of BBR for 16 weeks. Parameters, including hepatic fat content, serum glucose level, serum lipid profiles, liver enzymes, and serum and urine BBR concentrations, were measured before and after treatment. LSI and BBR showed a reduction in hepatic fat content as compared to LSI and were better than pioglitazone in reducing body weight and resulted in better lipid profiles (Yan et al., 2015). Furthermore, a mechanism-based study revealed that BBR reduced hepatic TG accumulation and decreased the expressions of hepatic stearyl-coenzyme A desaturase 1 (SCD1) and other TG synthesis-related genes (Zhu et al., 2019). Berberine administration was also reported to recruit and activate BAT in both humans and mice (Wu et al., 2019).

## Conclusion

Although there are many effective therapeutic drugs for the treatment of metabolic diseases, the current treatment did not control the rapid increasing trend in diabetes mortality and morbidity. Various therapeutic agents from both natural and synthetic sources are being investigated in patients with clinical signs of diabetic and other metabolic diseases. Formulations prepared from the various plant parts of *Berberis* species were found to be used traditionally in the treatment of diabetes and other metabolic diseases and related complications. A review of the scientific literature revealed that the extracts, isolated alkaloids from *Berberis* species including BBR and their derivatives, have shown promising effects in the studies related to diabetes and other metabolic diseases. The relatively low cost of BBR or supplements or extracts containing BBR, compared to other synthetic medications, will be of an advantage to the patients living in developing countries with poor socioeconomic circumstances. However, currently available scientific evidence is still not fully sufficient to prove their efficacy clinically. Further randomized double-blind clinical trials with a large number of patients and standardized clinical assessments are required to prove the effectiveness of the *Berberis* extracts and isolated compounds on metabolic diseases alone or in combinations. Novel pharmacological assessment techniques and analytical techniques will further provide additional opportunities for these agents. Moreover, the development of novel formulations of berberine could be an effective strategy for increasing its effectiveness against diabetes and other metabolic diseases.

## Author Contributions

TB, IB and JE conceptualized the manuscript. TB, AB, HD, HU, HK, IB and JE wrote the initial manuscript. TB, HD, HU, AP, IB and JE revised the manuscript. All authors agreed on the final version of the manuscript.

## Conflict of Interest

The authors declare that the research was conducted in the absence of any commercial or financial relationships that could be construed as a potential conflict of interest.

## Abbreviations

2h-PPG, 2-hour postprandial plasma glucose; A-FABP, Adipocyte fatty acid–binding protein; ALT, alanine aminotransferase; AST, aspartate aminotransferase; AMPK, AMP-activated protein kinase; BBA, berbamine; BBD, benign breast disease; BBR, berberine; BFFAL, blood free fatty acids level; BF, berberine fumarate; BGL, blood glucose levels; BJ, Berberis juice; b.i.d., twice daily; BMI, body mass index; BP, blood pressure; BW, body weight; BWG, body weight gain; CAT, catalase; C/EBPα, CCAAT enhancer binding protein alpha; CMS, cardio metabolic syndrome; COX2, Cyclooxygenase-2; CPK, serum creatine phosphokinase; DAG, diacylglycerol; DBP, diastolic blood pressure; DVIS, diabetic vascular insulin sensitivity; DPP-IV, dipeptidyl-peptidase IV; DN, Diabetic nephropathy; eNOS, endothelial nitric oxide synthase; EZE, ezetimibe; FA, fructosamine; FASN, fatty acid synthase; FBS, Fasting Blood Sugar; FBGL, fasting blood glucose levels; FOP, fibrodysplasia ossificans progressive; FPG, fasting plasma glucose; FPI, fasting plasma Insulin; FSIL, fasting serum insulin level; GHb, glycosylated hemoglobin; GLP-1, glucagon-like peptide-1; GLUT4, Glucose transporter type 4; GPx, glutathione peroxidase; GR, glutathione reductase; HbA1c, glycated haemoglobin; HDL-C, high density lipoprotein HDL-cholesterol; HFD, High Fat Diet; HOMA-R, Homeostatic Model Assessment; HOMA-IR, and HOMA-β%; IC, insulin concentration; IFG, Impaired fasting glycemia; IL-6, Interleukin-6; iNOS, Inducible nitric oxide synthase; INSR-mRNA, insulin receptor gene messenger RNA; InsR, Insulin resistance; InsS, insulin sensitivity; LDL-C, low density lipoprotein cholesterol; LDLR, Low density lipoprotein receptor; LEL, liver enzyme levels; Lp, lipid profile; MALA, metformin-associated lactic acidosis; MAPK, Mitogen activated protein kinase; MDA, Malondialdehyde; MMP, mitochondrial membrane potential; MS, metabolic syndrome; RCT: randomized, controlled trial; OXPHOS, impaired oxidative phosphorylation; PAB, prooxidant-antioxidant balance; pBBR, pseudoberberine; PG, plasma glucose; PGs, prostaglandins; P-gp, P-glycoprotein; pi3k, phosphoinositol 3 kinase; PKC, protein kinase C; PMBG, post-meal blood glucose; PON1, Paraoxonase-1; PPARα, peroxisome proliferator activated receptor alpha; PPARγ, peroxisome proliferator activated receptor gamma; PPBG, postprandial blood glucose; SBP, systolic blood pressure; SOD, superoxide dismutase; SREBP-1, sterol regulatory element-binding protein 1; STZ, streptozotocin; T1DM: type-1 diabetes mellitus; T2DM, type-2 diabetes mellitus; TAG, triacylglycerol; TC, total cholesterol; TG, triglycerides; TIC, total insulin consumption; TIS, total insulin secretion; TLR4, Toll-like receptor 4; TNFα, tumor necrosis factor alpha; ULK1, Unc-51-like autophagy-activating kinase 1.
